# Plant richness, land use and temperature differently shape invertebrate leaf-chewing herbivory on plant functional groups

**DOI:** 10.1007/s00442-022-05199-4

**Published:** 2022-06-17

**Authors:** Ute Fricke, Sarah Redlich, Jie Zhang, Cynthia Tobisch, Sandra Rojas-Botero, Caryl S. Benjamin, Jana Englmeier, Cristina Ganuza, Rebekka Riebl, Johannes Uhler, Lars Uphus, Jörg Ewald, Johannes Kollmann, Ingolf Steffan-Dewenter

**Affiliations:** 1 Department of Animal Ecology and Tropical Biology, Biocenter, University of Würzburg, Würzburg, Germany; 2 Institute for Ecology and Landscape, Weihenstephan-Triesdorf University of Applied Sciences, Freising, Germany; 3 Restoration Ecology, TUM School of Life Sciences, Technical University of Munich, Freising, Germany; 4 TUM School of Life Sciences, Technical University of Munich, EcoclimatologyFreising, Germany; 5 Field Station Fabrikschleichach, Department of Animal Ecology and Tropical Biology, University of Würzburg, Würzburg, Germany; 6 Professorship of Ecological Services, Bayreuth Center of Ecology and Environmental Research (BayCEER), University of Bayreuth, Bayreuth, Germany

**Keywords:** Climate, Ecosystem function, Land use, Plant guilds, Plant–insect interactions

## Abstract

Higher temperatures can increase metabolic rates and carbon demands of invertebrate herbivores, which may shift leaf-chewing herbivory among plant functional groups differing in C:N (carbon:nitrogen) ratios. Biotic factors influencing herbivore species richness may modulate these temperature effects. Yet, systematic studies comparing leaf-chewing herbivory among plant functional groups in different habitats and landscapes along temperature gradients are lacking. This study was conducted on 80 plots covering large gradients of temperature, plant richness and land use in Bavaria, Germany. We investigated proportional leaf area loss by chewing invertebrates (‘herbivory’) in three plant functional groups on open herbaceous vegetation. As potential drivers, we considered local mean temperature (range 8.4–18.8 °C), multi-annual mean temperature (range 6.5–10.0 °C), local plant richness (species and family level, ranges 10–51 species, 5–25 families), adjacent habitat type (forest, grassland, arable field, settlement), proportion of grassland and landscape diversity (0.2–3 km scale). We observed differential responses of leaf-chewing herbivory among plant functional groups in response to plant richness (family level only) and habitat type, but not to grassland proportion, landscape diversity and temperature—except for multi-annual mean temperature influencing herbivory on grassland plots. Three-way interactions of plant functional group, temperature and predictors of plant richness or land use did not substantially impact herbivory. We conclude that abiotic and biotic factors can assert different effects on leaf-chewing herbivory among plant functional groups. At present, effects of plant richness and habitat type outweigh effects of temperature and landscape-scale land use on herbivory among legumes, forbs and grasses.

**Supplementary Information:**

The online version contains supplementary material available at 10.1007/s00442-022-05199-4.

## Introduction

Anthropogenic land use and climate change pose threats to biodiversity with consequences for ecosystem functioning (Oliver et al. 2015). An important ecosystem function, which facilitates energy flux from primary producers to higher trophic levels, is herbivory (Cebrian and Lartigue 2004; Turcotte et al. 2014). In many ecosystems, insect herbivores are among the major contributors to herbivory and play a key role in food webs and nutrient cycles (Schowalter 2016). Land use and climate change may affect herbivory, as they shape species composition and physiology of invertebrate herbivores, but also of their interaction partners such as plants and predators (Rosenblatt and Schmitz 2016; Díaz et al. 2019; Martin et al. 2019). Yet, large-scale experimental studies on individual and interactive effects of temperature, plant richness and land use factors on invertebrate herbivory are lacking, though important for identifying factors, which possibly buffer effects of higher temperatures on herbivory.

Invertebrate herbivory of a plant strongly depends on its nutritious quality and palatability (Loranger et al. 2012; Njovu et al. 2019), which varies substantially among plant functional groups, namely legumes, non-leguminous forbs and grasses (Scherber et al. 2006). Legumes contain more nitrogen, e.g., higher crude plant protein content and lower leaf C:N ratio, than forbs and grasses (Perez Corona et al. 1995; Leingärtner et al. 2014), whereas silica acts as feeding deterrent in grasses (Massey et al. 2006). Consequently, from legumes to non-leguminous forbs and grasses in general, a decreasing intensity of leaf-chewing herbivory intensities is observed (Scherber et al. 2006; Leingärtner et al. 2014).

At the same time, insect herbivory is also strongly affected by abiotic factors, in particular temperature (Bale et al. 2002). Elevated temperatures affect herbivores and their interaction partners, i.e., plants and predators, in multiple ways, including alterations in nutrient requirements, development time and interactions between them (Bale et al. 2002; Rasmann and Pellissier 2015; Rosenblatt and Schmitz 2016). Over time, this can lead to local extinction of species and shifts in their geographic distribution (Bale et al. 2002; Thomas et al. 2004; Rasmann and Pellissier 2015), resulting in altered plant, herbivore and predator communities in a habitat patch. As herbivores are regulated both by resource availability (bottom-up) and predators (top-down) (Barnes et al. 2020), different temperature effects at multiple levels of the trophic cascade can lead to increased, decreased or unchanged herbivory by invertebrates (Rosenblatt and Schmitz 2016), while the pattern may differ among plant functional groups with largely differing C:N ratios. For instance, when temperatures rise, the metabolic rates of invertebrate herbivores may increase and cause a shift in the diet of generalist herbivores towards plants with higher C:N ratio (Rosenblatt and Schmitz 2016; Schmitz et al. 2016), such as from legumes to grasses or non-leguminous forbs. Thus, studying herbivory among plant functional groups may provide novel insights into the effects of temperature.

However, adverse temperature effects on ecosystem functions may be buffered in more diverse herbivore communities (Oliver et al. 2015). Herbivore richness and abundance (Ebeling et al. 2014; Schuldt et al. 2019), and possibly invertebrate herbivory (Ebeling et al. 2014; Meyer et al. 2017), are favored by higher plant species richness. On the other hand, plant species richness can also decrease herbivory (Unsicker et al. 2006; Jactel and Brockerhoff 2007), as specialist invertebrate herbivores, which often feed within a plant genus or family (Haddad et al. 2001), are less likely to encounter their host plant and to form dense populations in more diverse patches (Root 1973). Therefore, diverse plant communities comprising more taxonomic distant species can result in a reduction of herbivory (Jactel and Brockerhoff 2007), thereby potentially counteracting processes where plant species richness increases herbivory (Dinnage 2013).

Besides plant richness characterizing the local habitat and shaping the local invertebrate community, habitat amount is relevant to sustain high species richness (MacArthur and Wilson 1963; Fahrig 2013), e.g., of invertebrate herbivores. For instance, for a herbivore community on a local patch of herbaceous vegetation, the amount of available habitat can be approximated as the proportion of managed grassland in the area. However, the accuracy of this approximation depends on the plant functional group, as grasses are more commonly present on managed grasslands than legumes. Thus, with increasing grassland proportion, herbivory may increase more strongly on grasses than legumes. This increase of invertebrate herbivore richness with larger habitat amount (MacArthur and Wilson 1963; Fahrig 2013) could modulate temperature effects on herbivory (Oliver et al. 2015).

Open herbaceous vegetation occurs as part of grasslands or adjacent to other habitats such as forests (e.g., clearing), arable fields (e.g., field margin) and settlements (e.g., parks). The habitat type adjacent to a patch of open herbaceous vegetation may affect the available amount of habitat (e.g., open herbaceous vegetation), habitat isolation as well as the herbivore community composition. For example, forests can constitute barriers to dispersal of invertebrate herbivores (Schmitt et al. 2000), which can lead to species impoverishment in small herbaceous patches embedded in forests (Rösch et al. 2013). Thus, herbivore communities may differ depending on the adjacent habitat type, which may result in differences in invertebrate herbivory, but also in the response of herbivory to temperature.

Diverse landscapes promote richness and abundance of generalist invertebrate herbivores (Jonsen and Fahrig 1997). Thereby, high landscape diversity (Shannon index) refers to the presence of more different habitat types, more similar proportions of habitat types or both. Generalist herbivores may benefit from more than one habitat type due to supplementary or complementary resource use, when moving between habitat types (Dunning et al. 1992). Thus, landscape diversity may be a better approximation of habitat availability to generalist herbivores. Increases in generalist richness and abundance may increase herbivory. Therefore, diverse landscapes may indirectly modulate temperature effects on herbivory as well as increased proportions of generalists may favor temperature-induced shifts in herbivory from legumes (low C:N ratio) to grasses or forbs.

Here, we aim to disentangle the combined effects of temperature, plant richness and land use on invertebrate leaf-chewing herbivory among three plant functional groups. For this purpose, we studied herbivory on open herbaceous vegetation adjacent to typical habitat types in the temperate region (forest, grassland, arable field, and settlement) along large geographic gradients of local mean temperature, multi-annual mean temperature, plant richness at species and family level, and proportions of grassland and landscape diversity. In particular, we address the following questions:

1. How does temperature affect herbivory on three plant functional groups with largely differing C:N ratios?
2. How does plant richness and land use at multiple scales (habitat type, grassland proportion, landscape diversity) affect invertebrate herbivory among plant functional groups?
3. Do temperature and plant richness or land use interactively affect invertebrate herbivory on plant functional groups?

## Materials and methods

### Study area and plot selection

Research was conducted on 179 plots across Bavaria, Germany. To disentangle the combined effects of climate and land use on herbivory in three plant functional groups, we used a novel multi-scale study design which combined climate zones, regional land-use types, and a wide range of local habitat types (Redlich et al. 2021). Fifteen combinations of climate zones (multi-annual mean temperature between 1981 and 2010; < 7.5 °C, in 0.5 °C steps until 9 °C, > 9 °C) and regional land-use types (near-natural, agriculture and urban) were chosen from 5.8 km × 5.8 km grid cells covering Bavaria, each with four replicates (= 60 ‘regions’). Regional land-use types were defined by land cover: near-natural by > 85% near-natural vegetation with a minimum of 50% forest, agriculture by > 40% arable land and managed grassland, and urban by > 14% housing, industry and traffic infrastructure. In each region, plots were placed in the three dominant out of four possible habitat types (forest, grassland, arable field, settlement), and in the more contrasting habitat types if regional land cover was similarly distributed among habitat types. Additional plot selection criteria were avoiding overlap of 1 km ‘buffer zones’ among plots and keeping at least 50 m distance to larger roads and other habitat types (Redlich et al. 2021). Plots were established as 30 m × 3 m strips on open herbaceous vegetation, such as forest glades and clearings, grazed, mown and mulched grasslands, field margins and grasslands in proximity to crop fields, and parks and meadows within settlement areas.

### Assessment of herbivory by leaf-feeding invertebrates

Aboveground invertebrate herbivory was measured in the plots once in the period from end-May until mid-June 2019 (spring season). We assessed the dominant leaf damage type, with respect to damaged leaf area proportion by invertebrates from different feeding guilds (chewer, sucker, miner, unknown) for three herbaceous plant functional groups: legumes, non-leguminous forbs (following ‘forbs’) and grasses (Table S1). Chewing leaf damage dominated across plant functional groups, supporting the importance of this study. To refer to herbivory by leaf-chewing invertebrates, we use in the following the terms ‘herbivory’ and ‘leaf area loss’ interchangeably. We quantified proportional leaf area loss to leaf-chewing invertebrates for the above mentioned three herbaceous plant functional groups. Legumes contained representatives of the plant family Fabaceae only. Forbs encompassed species of various herbaceous angiosperm families except for the plant family Fabaceae and for plant families within the order Poales. Grasses included graminoids of the plant family Poaceae and occasionally species of the Cyperaceae family. These three plant functional groups are commonly distinguished and differ largely in several traits, particularly in C:N ratio and protein content (Perez Corona et al. 1995; Leingärtner et al. 2014), and commonly differ in herbivory levels (Scherber et al. 2006; Leingärtner et al. 2014).

Per plant functional group, three plant individuals of three ‘plant species’ were haphazardly selected for the collection of three leaves (total of 27 plant individuals and 81 leaves per plot). This approach assured that multiple plant species were sampled within plant functional groups, but due to the large number of fieldworkers involved in this project, this was done based on morphological traits without determining individual species identity. Therefore, we use quote marks to refer to ‘plant species’ in the context of our herbivory assessment. The plant species list provided as supporting information (Table S2) was based on separate vegetation surveys, and were only available after the leaf sampling. Since no single plant species occurred across all plots, e.g., the third most frequent legume species occurred in only 46 out of 179 plots (Table S2), ‘plant species’ assessed for leaf-chewing herbivory differed among plots. Due to the haphazard selection of ‘plant species’, abundant species within plant functional groups were more likely to be sampled.

From each individual plant, leaves from the apical, middle and basal nodes—in case of grasses, top, middle and bottom blade on the stem of tillers—were pinched off, mounted in a notebook with transparent tape, pressed and dried for later assessment of leaf damage. Both leaf position as selection criterion and digital assessment in the lab were chosen to minimize observer bias and also to include leaves of different ontogenetic stages that may be disproportionately affected by herbivory (Sand-Jensen et al. 1994). Leaf-chewing herbivory was higher on basal than apical, aka older than younger, leaves across plant functional groups (Table S1).

Proportional leaf area loss was determined using the BioLeaf app (Machado et al. 2016), which automatically transformed color images to binary images (only black and white pixels) and calculated proportional leaf area loss based on white parts enclosed by black leaf area. Therefore, some prior image adjustments were needed: (1) Nibbled leaf margins were straightened or adjusted to restore the pre-damage leaf contour with a thin black line in order to capture nibbled leaf margins as missing leaf area; and (2) overlapping leaf parts were separated with a thin white line connecting the white space to the surroundings of the leaf to not falsely be assigned as missing leaf area by the Bioleaf app. Images were adjusted using GIMP software (The GIMP Development Team 2017).

### Measures of plant richness

Vegetation surveys were conducted between May and July 2019 (seven subplots on each plot, adding up to 10 m^2^ total sampling area per site). Recorded plant species and families were summed up per plot to achieve plant richness at species and family level. Ferns, horsetails and woody plants as part of the herb layer were considered for total plant richness measures but not for herbivory assessment. A list of plant species and families present on plots is provided in Table S2.

### Measures of land use at multiple spatial scales

Local similarities among plots of open herbaceous vegetation were captured by the predictor ‘habitat type’, which denotes the adjacent habitat to the plots, i.e., forest, grassland, arable field and settlement.

As landscape predictors, we considered landscape diversity and proportion of grassland at multiple scales around the center of the plots (0.2 km, 0.5–3.0 km in 0.5 km steps; seven spatial scales). Landscape diversity was calculated as Shannon Index from detailed land-cover maps distinguishing six land-use categories: natural/semi-natural, forest, grassland, arable, urban and water (combination of ATKIS 2019, CORINE 2018 and IACS 2019; for details see Fig. S1). Proportion of grassland mirrors the proportion of the respective land-use category.

### Measures of temperature

Local mean temperatures were derived from thermologgers (ibutton, type DS1923) attached to the north side of wooden poles, at 1.1 m above ground and roughly 0.15 m below a wooden roof, preventing direct solar radiation. We established one thermologger per plot and extracted the local mean temperature during the study-site specific 1 month period prior to leaf sampling from hourly temperature measurements.

We retrieved 30 year multi-annual mean temperatures per plot based on gridded monthly averaged mean daily air temperatures with a horizontal resolution of 1 km from 1981 to 2010 (Deutscher Wetterdienst 2020). This climate variable was chosen to study climate and land-use effects in a space-for-time framework (Blois et al. 2013; Redlich et al. 2021).

### Data analysis

Data on proportional mean leaf area loss to chewing invertebrates were averaged per plant individual, ‘plant species’ and plant functional group for each plot to equally account for individuals and species despite missing leaves and plant individuals. Sampling of different plant species was assured due to morphological traits. As we did not intend to conduct a plant species-specific assessment of the leaf-chewing herbivory data, exact plant species identity was not determined. Averaging leaf area loss per plot was favored over a multiple-nested random term accounting for nested sampling structure to avoid model fitting issues related to missing values and information, e.g., missing recordings of leaf position or missing leaves due to processing damage. We also excluded data from all plots of which we obtained proportional mean leaf area loss data of ˂10 leaves of each plant functional group prior to herbivory analysis, to cover identical predictor ranges among plant functional groups. The application of exclusion criteria resulted in data from 80 plots in 39 regions being included in the analysis.

Invertebrate leaf-chewing herbivory data were analyzed with beta regression to cope with continuous proportional data (Yellareddygari et al. 2016; Douma and Weedon 2019). In preparation for beta regression, zeros were replaced with a small value (0.00001; slightly lower than the smallest value; Douma and Weedon 2019). Leaf damage by leaf-chewing invertebrates on legumes and forbs was absent on a single plot each, and was absent on grasses on two plots.

As candidate predictors, we included plant functional group, local mean temperature, multi-annual mean temperature, land use at local (habitat type) and landscape-scale (proportion of grassland area, landscape diversity; seven spatial scales in separate models), and local plant richness (species and family level). Predictor values were *z*-transformed prior to analysis, while the selected best models are presented with untransformed predictor variables. Ten separate models were created, each of them containing plant functional group, multi-annual mean temperature, one of the four land-use and plant-richness variables (at different spatial scales, if applicable) and all interactions up to the three-way interaction term. Separate models were preferred over one model containing all land-use and plant-richness variables to avoid over parameterization.

The model including the three-way interaction of plant functional group, multi-annual mean temperature and habitat type indicated a trend in grassland, which was further explored using a data subset of grassland plots. This was also done for comparison with other studies, as herbivory studies are commonly conducted on grassland. An additional model containing multi-annual mean temperature, habitat type and their interaction term, was fitted to the subset with the rest of the analysis approach being equal. For comparison, an additional model containing local mean temperature instead of multi-annual mean temperature was fitted to the grassland subset.

A nested random term for ‘plot’ in ‘region’ (three plots per region) was included to account for plant functional groups on the same plots and clustering of plots (Redlich et al. 2021). When grassland subsets were analyzed, only ‘plot’ was used as a random term. This nested random term was retained throughout the model selection process (Bolker et al. 2008).

The majority of maximum variance inflation factors were < 4, which falls below the commonly applied threshold for collinearity of variance inflation factor ˃ 10 (Chatterjee and Price 1991). Variance inflation factor exceeded the threshold in some models containing interaction terms with habitat type. Additionally, a correlation matrix of continuous predictor variables was calculated (Table S3) and continuous predictors were plotted by habitat type (Fig. S2) to visually assess relationships between continuous and categorical predictor variables. Continuous predictors were not or only weakly correlated except for a strong positive correlation between plant richness at species and family level (Pearson’s *r *= 0.76, *P* < 0.001, Table S3). For a comparison of continuous predictor ranges among habitat types see Fig. S2.

Models with all possible predictor combinations were compared by the goodness of fit based on Akaike’s information criterion corrected for small sample size (AICc). The lower AICc, the better the relative goodness of model fit. Competing multivariate models with a difference of less than two (∆AICc ˂ 2) were considered equal (Burnham and Anderson 2002), and then the more parsimonious model was chosen. Model selection of fixed effects (predictors) was done with models fitted by maximum likelihood, while the selected best model was fitted and is presented by restricted maximum likelihood (Zuur et al. 2009). Tukey post hoc analysis was used to compare herbivory between levels of categorical variables (i.e., plant functional groups, habitat types) and to correct for multiple comparisons.

To gain further insights on the relative importance (sum of Akaike weights) of the candidate predictors for herbivory of the single plant functional groups, multimodel averaging was conducted for each plant functional group separately, including plant richness either at species or family level (Fig. S3 + text).

Data analysis was done with R version 4.0.3 (R Core Team 2020) using the packages ‘glmmTMB’ (Brooks et al. 2017), ‘emmeans’ (Russell 2020), ‘Hmisc’ (Harrell 2020), ‘MuMin’ (Barton 2020), ‘ggeffects’ (Lüdecke 2018), ‘DHARMa’ (Hartig 2020) and ‘performance’ (Lüdecke et al. 2020).

## Results

### Effects of plant richness and land use on herbivory among plant functional groups

**Fig. 1:**
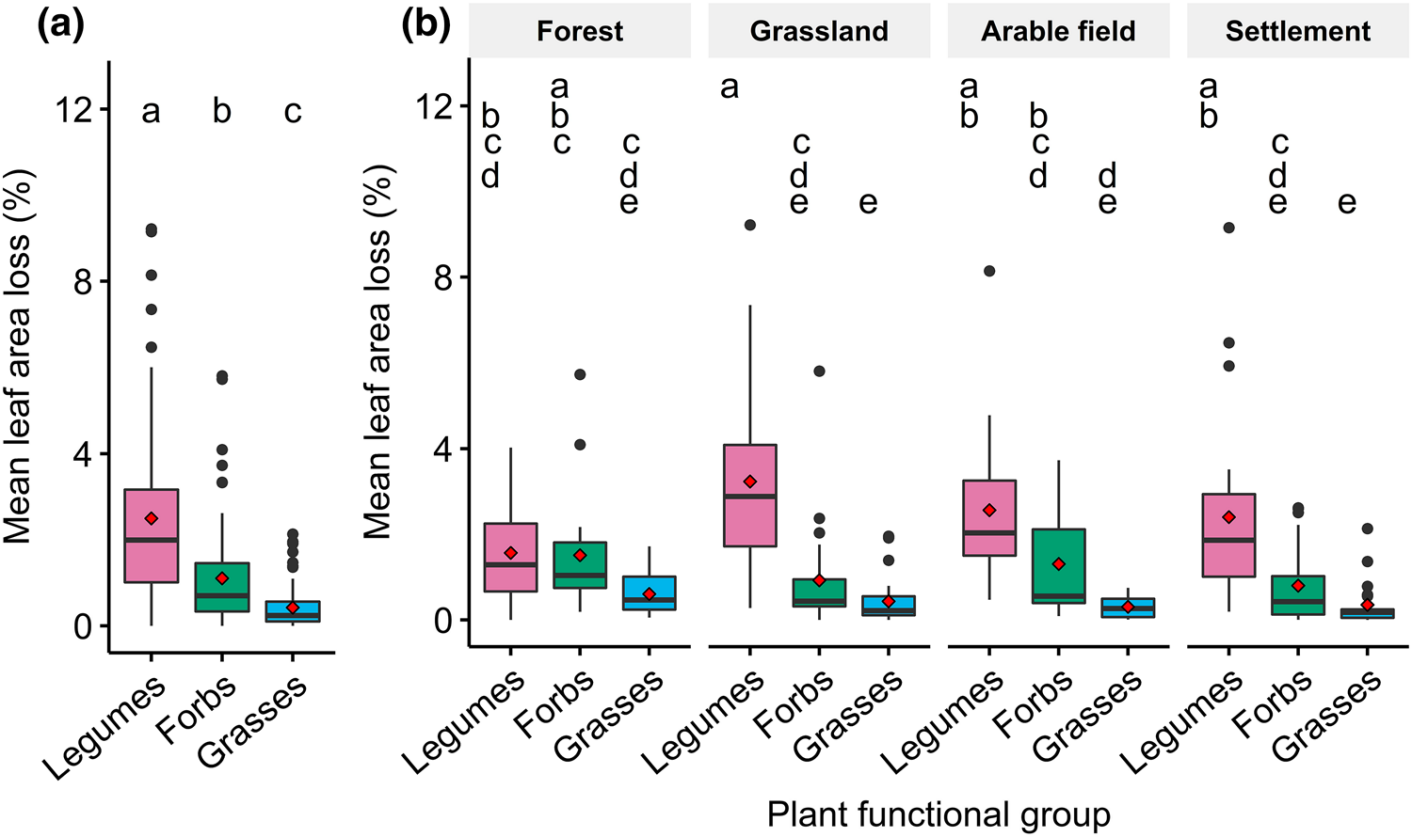
Effects of plant functional group **a** and interactive effects of plant functional group and habitat type **b** on mean leaf area loss to chewing invertebrates per plot. Red diamonds highlight mean values per plant functional group. Different lower case letters indicate differences between habitat types and plant functional groups evaluated by post hoc tests with Tukey correction after evaluation of the overall effects in beta regression models by ∆AICc and parsimony

Damage by leaf-chewing invertebrates on legumes, forbs and grasses ranged between 0 and 83, 0 and 59, and 0 and 19% area loss of individual leaves, respectively (Fig. S4). Among plant functional groups, plot-averaged leaf area loss on legumes (2.5%) was on average 2.3 times higher than on forbs (1.1%) and 5.9 times higher than on grasses (0.4%; Fig. 1a). This pattern was mirrored in most habitats except forests, where herbivory was similar across plant functional groups and herbivory on legumes was lower than in grassland (Fig. 1b) Herbivory on forbs and grasses was not substantially different among habitat types.

**Fig. 2:**
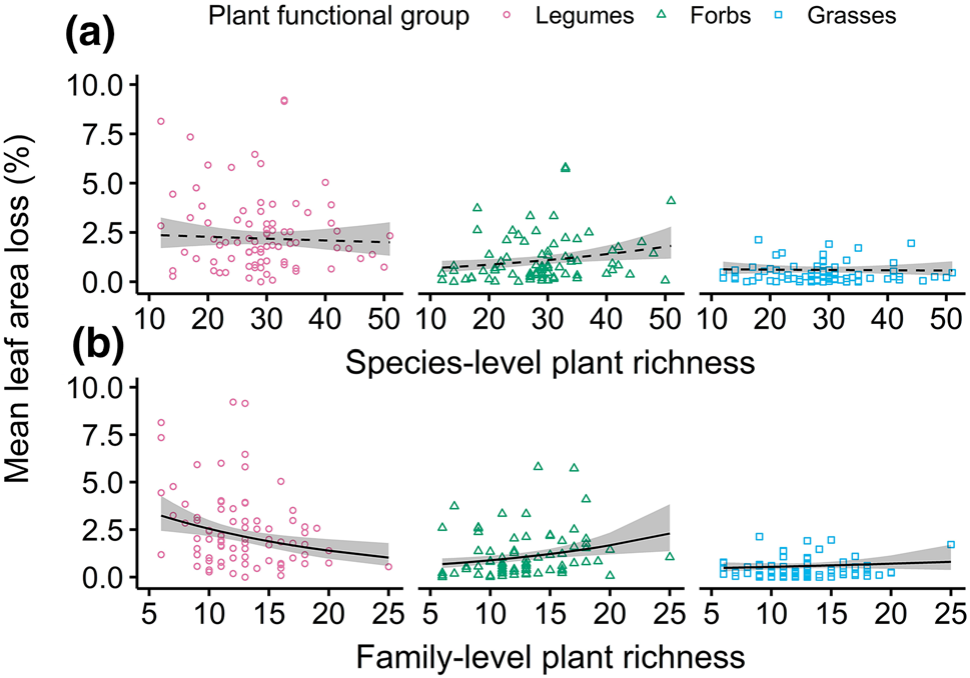
Interactive effects of plant richness with plant functional group (legumes: pink circles, non-leguminous forbs: green triangles: grasses: blue squares) on plot-averaged leaf area loss to chewing invertebrates. Panels show interactive effects with **a** plant richness at species level and **b** family level. Lines present predictions of full beta mixed models (solid when interaction term supported, else dashed). Gray shades indicate 95% confidence bands. Model selection was based on ∆AICc and parsimony

Invertebrate leaf-chewing herbivory did not depend on plant richness at species level (Fig. 2a, Table S4 + 5), but with increasing total numbers of plant families, herbivory on legumes decreased while herbivory on forbs and grasses increased (Fig. 2b). As plant richness at family level was higher in forests than in other habitat types (Fig. S2), the differential effects of habitat type and of family-level plant richness among plant functional groups cannot be clearly separated. However, when assessing the relative importance of all candidate predictors on leaf-chewing herbivory separately per plant functional group means multimodel averaging, and including plant richness at species and family level in separate models (Table S3). Habitat type was the relatively most or second most important predictor of herbivory on all three plant functional groups (Fig. S3). Relative importance of habitat type was especially high (Σwi ≥ 0.8) for herbivory on legumes, and on forbs and grasses only when family-level plant richness was not included. Family-level plant richness was the relatively most important predictor (Σwi = 0.7) or second most important predictor (Σwi = 0.4) for leaf-chewing herbivory on forbs and legumes, respectively, but not for grasses (Fig. S3). Thus, habitat type was relatively more important than family-level plant richness for legume herbivory, but not for herbivory on forbs. Nonetheless, the pattern of decreasing leaf-chewing herbivory on legumes towards higher plant richness at family level seemed to persist across habitat types (Fig. S5), which supports a weak effect of plant richness at family level also on legumes.

At multiple spatial scales, invertebrate herbivory among plant functional groups was similar across the observed ranges of proportions of managed grassland and landscape diversity (Table S4 + 5, Fig. S3).

### Interactive effects of temperature and land use on herbivory of plant functional groups

**Fig. 3:**
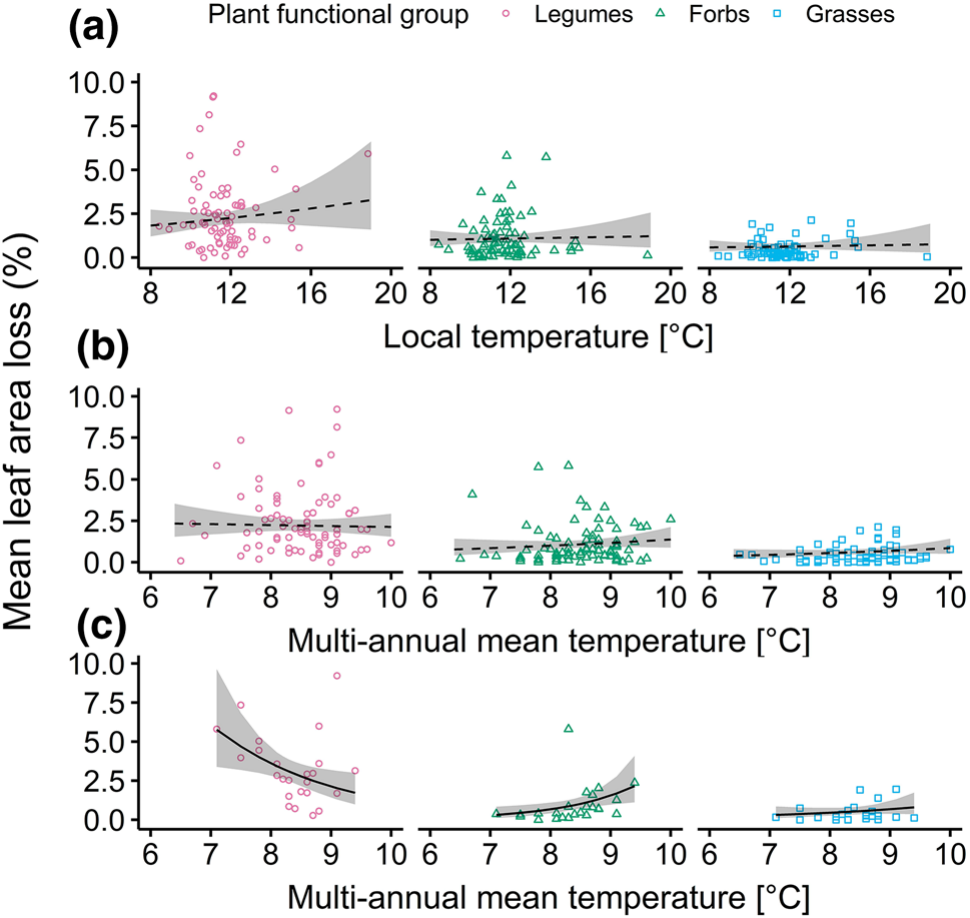
Interactive effects of temperature with plant functional group (legumes: pink circles, non-leguminous forbs: green triangles: grasses: blue squares). Panels show interactive effects with **a** local mean temperature (80 plots), **b** multi-annual mean temperature (80 plots) and **c** multi-annual mean temperature including grassland plots only (24 plots). Lines indicate predictions of the full beta mixed model (solid when interaction supported, else dashed) based on the complete data set (**a**, **b**) or the grassland subset (**c**). Model selection was done using ∆AICc and parsimony

Both local mean temperature of the 1 month period prior to leaf sampling (Fig. 3a) and multi-annual mean temperature (Fig. 3b) did not substantially affect mean herbivory among plant functional groups (Table S4 + 5).

Three-way interactions of plant functional group, any of the temperature variables and single land-use or plant-richness predictors were not supported by ∆AICc and parsimony (Table S4 + 5). Yet in grassland plots, herbivory on legumes, forbs and grasses decreased, increased and slightly increased with higher multi-annual mean temperature, respectively (Fig. 3c,) Fig. S6, Table S6). However, local mean temperature did not affect herbivory among plant functional groups in grassland plots (Fig. S7, Table S6).

## Discussion

We compared invertebrate leaf-chewing herbivory—the dominant type of leaf damage in our study—among three major plant functional groups across geographic gradients of plant richness, land use and temperature, and elucidated the potential of biotic conditions to modulate temperature effects on herbivory. Herbivory differed among plant functional groups, and among plant functional groups in response to local habitat types and plant richness at family level, but showed no general response to plant richness at species level, proportion of grassland, landscape diversity, local mean temperature and multi-annual mean temperature, at the studied gradients. We found a differential effect of multi-annual mean temperature among plant functional groups in grassland plots (grassland subset). In the following, we discuss the presence or absence of differential temperature, plant richness and land use effects among plant functional groups, future research directions arising from our study and potential consequences of global warming on invertebrate leaf-chewing herbivory.

Plant richness at family level decreased invertebrate leaf-chewing herbivory on legumes and increased herbivory on non-leguminous forbs and (slightly on) grasses. In this study, higher family-level plant richness implies more plant families other than legumes (Fabaceae) and grasses (Poaceae), e.g., more plant families of forbs, horsetails, ferns and woody seedlings (part of the herb layer, particularly in forests). The observed pattern in herbivory suggests that invertebrate herbivores feeding on legumes are more negatively impacted by the presence of more plant families compared to more plant species, and that herbivores on legumes are more affected than those feeding on grasses. The first could result from herbivorous invertebrates often being specialized on feeding within plant families (Haddad et al. 2001), e.g., on legumes (Fabaceae), and from a reduced likelihood that a specialized herbivore will find and build-up high population densities on its host plants in diverse vegetation (Root 1973). Herbivory on grasses may respond less to plant richness compared to legumes for several reasons: (1) despite the higher plant richness, the proportion of grasses in the community may have remained high, (2) grasses may be more prone to generalist rather than specialist chewing invertebrates, which depend less on plant richness (Shinohara and Yoshida 2021), and (3) proportional leaf area loss to chewing invertebrates was generally very low on grasses compared to legumes with a much larger range of leaf area losses (see also Leingärtner et al. 2014), which means that any change in herbivory on grasses results in a small effect. The increased herbivory on forbs (functional group rich in plant families) towards higher plant richness at the family level may result from an increased likelihood of palatable plant families being among the forb species on a plot and being sampled (sampling effect). Thus, albeit community-level herbivory may increase with plant species richness (Ebeling et al. 2014; Meyer et al. 2017), herbivory on individual plant families may decrease, and even more strongly with plant richness at family level.

The proportion of grassland did not affect herbivory on any plant functional group. Larger proportions of grassland, and thus more habitat area of open herbaceous vegetation, was expected to increase herbivory, as species richness increases with increasing habitat amount (MacArthur and Wilson 1963; Fahrig 2013). The absence of an effect of grassland proportion may result from the measure of grassland proportion comprising managed grassland, but not all landscape elements of open herbaceous vegetation (forest clearing, parks, etc.). Besides, managed grasslands harbor different herbivore communities depending on the specific management (Shinohara et al. 2019). Therefore, the habitat amount available to a herbivore community may have diverged from the measured grassland proportion. Alternative explanations are that grassland proportion may have equally benefitted herbivores and predators, which canceled out grassland effects on herbivory, or that different herbivore communities can provide similar levels of herbivory (Rossetti et al. 2017). Although we did not observe an effect of grassland proportion on leaf-chewing herbivory, landscape elements may be relevant to herbivory, but their effect may only become visible using higher resolution maps, which better capture habitat amount (e.g., also forest clearings), and including measures on the herbivore and predator community.

Habitat type affected leaf-chewing herbivory among plant functional groups. Herbivory on legumes was lower in forests than in grasslands and intermediate in settlements and arable fields, and therefore herbivory was similarly low among plant functional groups in forests, compared to higher herbivory levels on legumes than on forbs and grasses in typical ‘open’ habitat types (grassland, arable field, settlement). The difference in herbivory on legumes between grasslands and forests cannot clearly be assigned to a single mechanism, but may result from lower habitat amount of open herbaceous vegetation in forests (Fahrig 2013), dispersal barriers constituted by forests (Schmitt et al. 2000) or both, reducing the probability of legume specialists to be present. Studies comparing herbivory on open habitat and inside forests also reported higher herbivory levels for open than forested habitats (Maron and Crone 2006; Dostálek et al. 2018). This may apply in particular to plant species or plant families that suffer from specialist herbivory and that are less prone to generalist herbivory, for example legumes (Fabaceae) compared to grasses (Poaceae). Common leaf-chewing generalist herbivores on open herbaceous vegetation are grasshoppers, which consume much more grasses than legumes (Unsicker et al. 2005). Thus, plant species of certain plant families may find refuge from invertebrate leaf-chewing herbivory in forests.

Landscape composition, here landscape diversity at various spatial scales (0.2–3.0 km), did not substantially affect invertebrate chewing herbivory among plant functional groups. Particularly generalist species benefit from diverse landscapes (Jonsen and Fahrig 1997). Thus, species richness of generalist herbivores may increase with landscape diversity at the expense of specialists, as communities tend towards equilibrium (MacArthur and Wilson 1963; Cazzolla Gatti 2016). Besides, differences in landscape diversity may also go along with more or less disturbance and higher or lower species richness and size of the herbivore community. However, a small number of common generalist herbivorous invertebrate species have the potential to maintain herbivory levels provided by more diverse herbivore communities (Rossetti et al. 2017). Thus, invertebrate herbivore community composition may have changed along the landscape diversity gradient without visible changes in invertebrate leaf-chewing herbivory.

Although warmer climates are expected to increase herbivory pressure (Rasmann and Pellissier 2015), we observed neither a general increase of invertebrate leaf-chewing herbivory in response to higher local mean temperature (1 month period prior to leaf sampling) nor to higher multi-annual mean temperature covered by our study design. However, in grassland plots herbivory on legumes decreased towards warmer climates, while herbivory increased on forbs and (slightly on) grasses. Why this pattern occurs only in grasslands cannot be clearly explained, but it may originate from differences in invertebrate communities among plots in different habitat types, which is suggested by differences in richness and biomass of flying insects among habitat types (Uhler et al. 2021). Differential responses of herbivory among plant functional groups in grasslands to multi-annual temperature, but not to local mean temperature, suggest temperature effects related to the herbivore community rather than to short-termed changes in herbivore physiology. However, more research will be needed to provide further evidence on differential rates of invertebrate leaf-chewing herbivory among plant functional groups (or plant families) towards higher temperatures and to identify the underlying mechanisms. Still, this observation in grassland plots provides further—albeit weak—evidence for differential responses in invertebrate herbivory among plant functional groups and habitat types, which should be considered in future studies (e.g., studying herbivory adjacent to different habitat types), as traditionally herbivory research is much focused on grasslands.

As the majority of plot-averaged leaf area losses to leaf-chewing invertebrates fell below 4% across our large climatic temperature gradient, it is unlikely that any other temperature measure not addressed in this study, elicited strong effects on herbivory under the studied conditions. However, herbivory on individual plant species or families—other than legumes (Fabaceae) and grasses (Poacea)—was not captured in this study, but may have responded more clearly to temperature. This is likely to be particularly true for plant species or families whose defences are highly temperature-sensitive or which are damaged by highly temperature-sensitive herbivores (reviewed in Rosenblatt and Schmitz 2016). Thus, albeit herbivory at the level of plant functional groups was not (or only in grasslands) affected by temperature, we cannot exclude that single plant species—e.g., relevant from a conservationist perspective—were not affected, particularly as we did observe proportional leaf area loss of up to 83% on individual leaves. Besides, herbivore communities may have adapted to the current temperature conditions over a long period of time, potentially contributing to similar herbivory levels across the studied multi-annual mean temperature gradient, but temperature effects may become apparent when global warming maintains its current pace and further exacerbates biodiversity loss (Thomas et al. 2004; Wagner 2020). With this study, we captured the current pattern of invertebrate leaf-chewing herbivory at the level of plant functional groups in response to a large multi-annual mean temperature gradient (6–10 °C), and found no evidence—except for grasslands—that leaf-chewing herbivory would be affected by higher temperatures.

The herbivory pattern among plant functional groups observed in this study—i.e., in response to family-level plant richness and habitat type—can be best explained through differences in legume specialists between those sites. Relationships of forb herbivory with plant richness, land use and temperature were much less clear, which may result from this plant functional group comprising multiple plant families. This emphasizes the relevance of studying herbivory on a taxonomic level, distinct from community-level herbivory. Our results suggest that the plant family level is suitable, e.g., as many herbivores are specialized within plant genus or family (Haddad et al. 2001). Besides, matching herbivory on a taxonomic level with measures of the herbivore community (e.g., richness and abundance of leaf-chewing herbivores feeding on a specific plant family) will likely provide valuable mechanistic insights into effects of plant richness, land use and temperature on herbivory.

## Conclusion

Overall plot-averaged herbivory by leaf-chewing invertebrates was rather low (< 4% leaf area loss) and often similar across the studied geographic gradients of abiotic and biotic factors (i.e., local mean temperature, grassland proportions, landscape diversity), suggesting that largely different herbivore communities provide similar levels of herbivory at plot level. However, invertebrate leaf-chewing herbivory among plant functional groups—particularly on single plant families (e.g., legumes)—responded differentially to plant richness at family level, land use (i.e., habitat type) and temperature (i.e., multi-annual mean temperature in grassland plots) across large geographic gradients, which may have consequences for the competitive relationships among plant families. This suggests that herbivory assessment at taxonomic level (e.g., plant families) provides more differential insights into responses of herbivory to biotic and abiotic factors than community-level herbivory. We found no evidence that local plant richness, habitat type, grassland proportion or landscape diversity modulate sensitivity of herbivory on three plant functional groups to temperature (e.g., indirectly via herbivore community), but also little evidence of both local mean temperature and multi-annual mean temperature effects on herbivory. Thus currently, effects of local plant richness and habitat type seem to be more relevant than temperature and landscape-scale land use to variation in invertebrate leaf-chewing herbivory among three plant functional groups.

## Supplementary Information

Below is the link to the electronic supplementary material.

Supplementary file1 (PDF 2974 KB)

## Data Availability

Data are available in the Dryad Digital Repository: 10.5061/dryad.4xgxd25cv.

## Code availability

R Code is available at Zenodo: 10.5281/zenodo.6637804.
