# Supplementary material for: Plant richness, land use and temperature differently shape invertebrate leaf-chewing herbivory on plant functional groups
Source: Oecologia. 2022 Jun 17;199(2):407–17. doi: 10.1007/s00442-022-05199-4 (PMC9225970; doi:10.1007/s00442-022-05199-4)
Supplement: Supplementary file 1 — Supplementary file1 (PDF 2974 KB) [file 442_2022_5199_MOESM1_ESM.pdf]

Electronic supplementary material

Plant richness, land use and temperature differently shape invertebrate leaf-chewing herbivory on plant functional groups

Ute Fricke*, Sarah Redlich, Jie Zhang, Cynthia Tobisch, Sandra Rojas-Botero, Caryl S. Benjamin, Jana Englmeier, Cristina Ganuza, Rebekka Riebl, Johannes Uhler, Lars Uphus, Jörg Ewald, Johannes Kollmann, Ingolf Steffan-Dewenter

*corresponding author: ute.fricke@uni-wuerzburg.de

Fig. S1 (+ Text) ………………………………………………………….. 2

Fig. S2 ……………………………………………………………......... 3

Fig. S3 (+ Text) …………………………………………………………… 4

Fig. S4 ……………………………………………………………......... 4

Fig. S5 ……………………………………………………………......... 6

Fig. S6 ……………………………………………………………......... 7

Fig. S7 ……………………………………………………………......... 8

Table S1 (+ Text) ………………………………………………………… 9

Table S2 ……………………………………………………………….. 11

Table S3 ……………………………………………………………….. 23

Table S4 ……………………………………………………………….. 24

Table S5 ……………………………………………………………….. 25

Table S6 ……………………………………………………………….. 26

Fig S1 + Text: Creating the detailed land-cover map

We distinguished six land-use categories within the state of Bavaria, i.e. natural/semi-natural habitat, forest, grassland, arable, urban and water, and created a detailed land-cover map based on these categories. For this purpose, we used three different data sources (IACS 2019, CORINE 2018, ATKIS 2019) that complemented each other and provided different levels of detail, as required for additional characterization of the landscape in further analysis (i.e. 'subcategories', not used here). We used ATKIS to define the boundary of different land-use types, IACS and CORINE to provide additional details for further characterization. In case of overlaps of data sources and land-use layers we prioritized specific land-use categories (semi-natural > forest > grassland > arable > urban > water) and data sources (see legend **Fig. S1**) to enhance spatial resolution and details related to subcategories used for additional spatial analysis. Land-use categories were then used to calculate landscape diversity and proportion of grassland around the center of each study site.

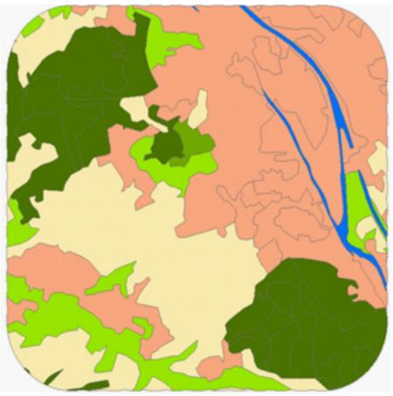

Land-use priority from top to bottom (and priority of data sources)

Semi-natural (CORINE subclass 'transitional woodland' > IACS > ATKIS)

Forest (ATKIS)

Grassland (IACS > ATKIS)

Arable (IACS > ATKIS)

Urban (ATKIS)

Water (ATKIS)

Gaps in map filled with CORINE

Fig. S1 Creation of detailed land-cover maps based on six main land-use categories within Bavaria (semi-natural habitat, forest, grassland, arable, urban, water). The map was created by combining three different land-cover maps (ATKIS 2019, IACS 2019, CORINE 2018).

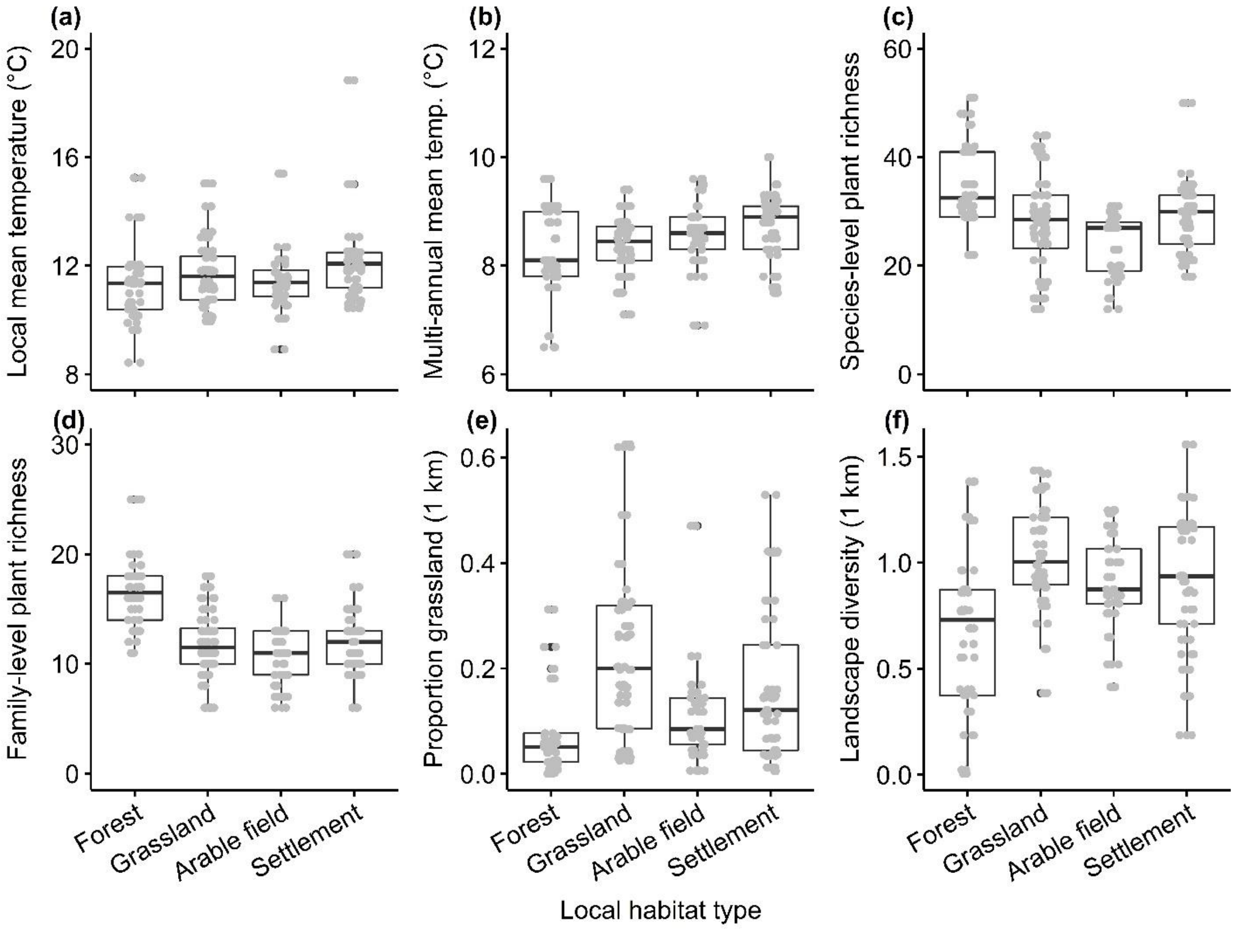

Fig. S2 Visual evaluation of relationships between the categorical predictor variable (habitat type) and the continuous predictor variables used for analysis of herbivory by leaf-chewing invertebrates (80 plots). (a) Local mean temperature (1-month period prior to leaf sampling, exception: 78 plots), (b) Multi-annual mean temperature, (c) Species-level plant richness (= total plant species richness), (d) Family-level plant richness (= total plant familial richness), (e) Proportion managed grassland in 1-km surrounding, (f) Landscape diversity at 1-km scale

Fig S3 + Text: Multimodel averaging per plant functional group

Models on plot-average leaf area loss to leaf-chewing invertebrates with all possible predictor combinations at each spatial scale were created separately per plant functional group, including either plant richness at species or family level due to high correlation (Pearson's r: 0.76, **Table S3**). Only region was included as a random term, as plot-averaged data of a single plant functional group was modelled. Models with all possible predictor combinations were obtained using the dredge function in the MuMIn package (Barton 2020). Then, the sum of Akaike weights (Σwi, range: 0–low to 1–high) was calculated of each spatial scale and of each predictor at every spatial scale. This value indicates the relative importance of a spatial scale (compared to other scales) and of a predictor variable (compared to the other predictors) at a certain spatial scale, respectively (Burnham and Anderson 2002).

References

Barton, K. (2020) MuMIn: Multi-model inference. Available at: https://cran.r-project.org/package=MuMIn.

Burnham KP, Anderson DR (2002) Model selection and multimodel inference: a practical information-theoretic approach, 2nd edn. Springer

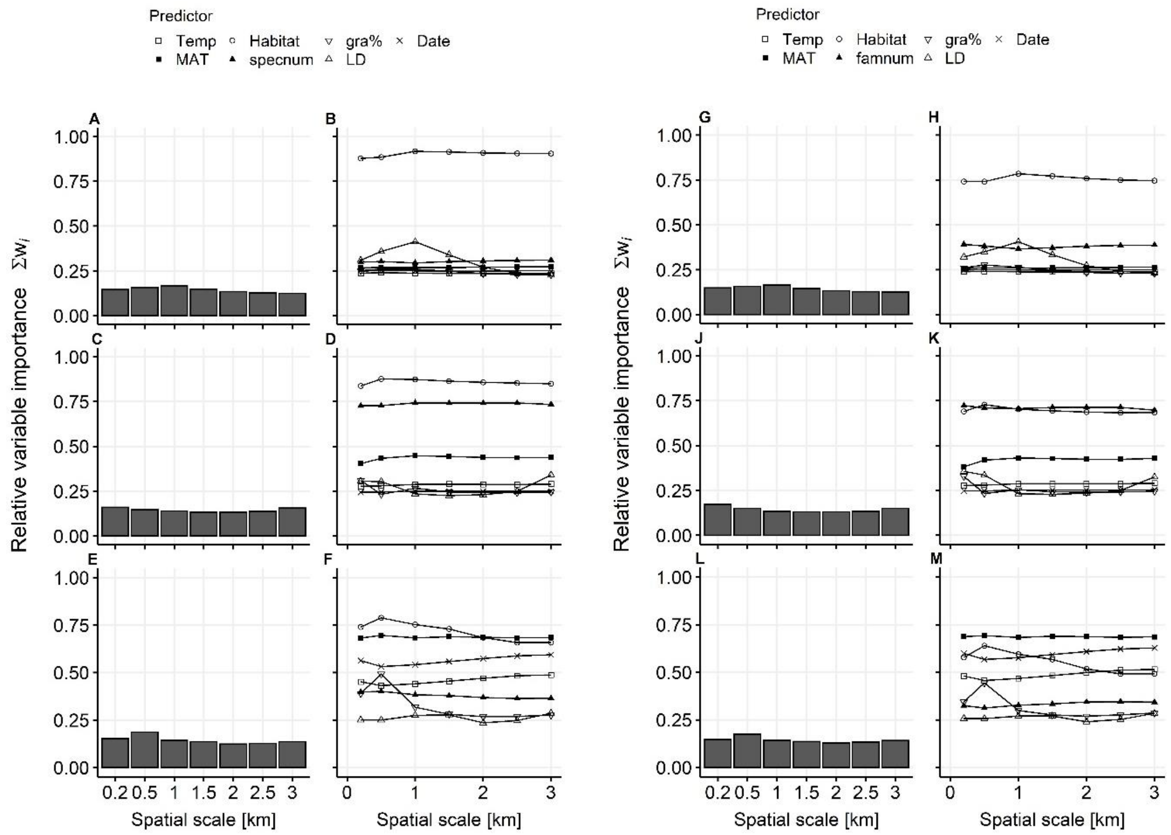

Fig. S3 Effects of candidate predictors (and leaf sampling date, 'Date') on leaf-chewing herbivory among three plant functional groups (upper row: legumes, middle row: forbs, bottom row: grasses, 78 plots); Relative importance of each spatial scale (0.2–3.0 km, bar graphs) and each predictor (line graphs); Values range between zero (low) and one (high); A–F) including plant species richness ('specnum'), G–M) including plant richness at family-level ('famnum'); Temp: Local mean temperature, MAT: Multi-annual mean temperature, Habitat: Adjacent habitat type, gra%: Proportion grassland, LD: landscape diversity

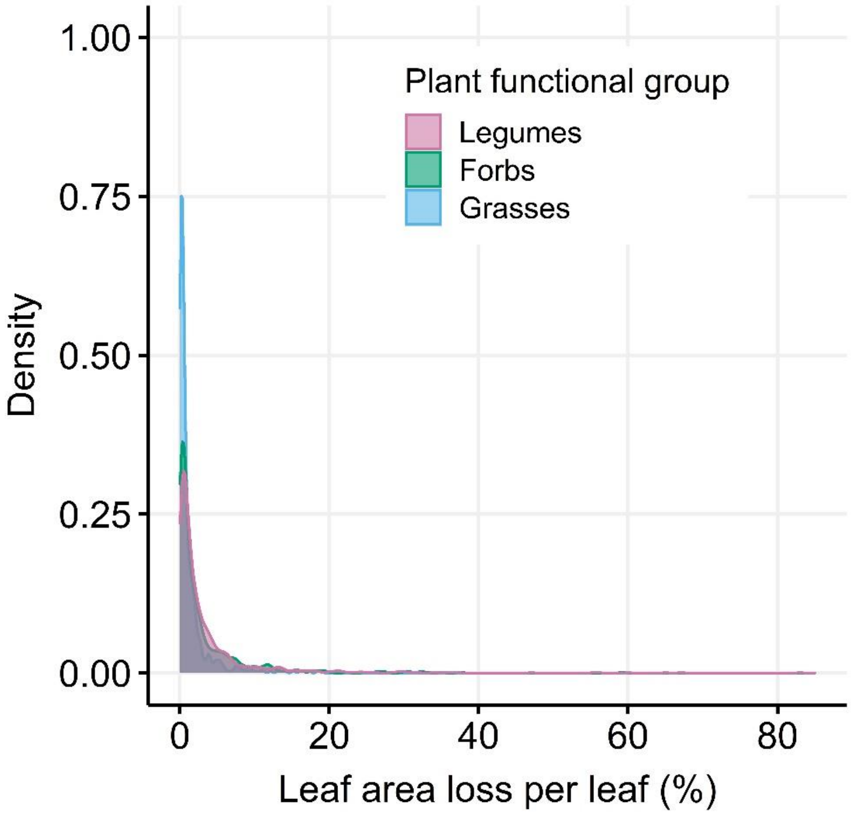

Fig. S4 Density distribution of proportional leaf area loss to chewing invertebrates on individual leaves, on which damage is present (> 0%), per plant functional group (80 plots, 2737 leaves).

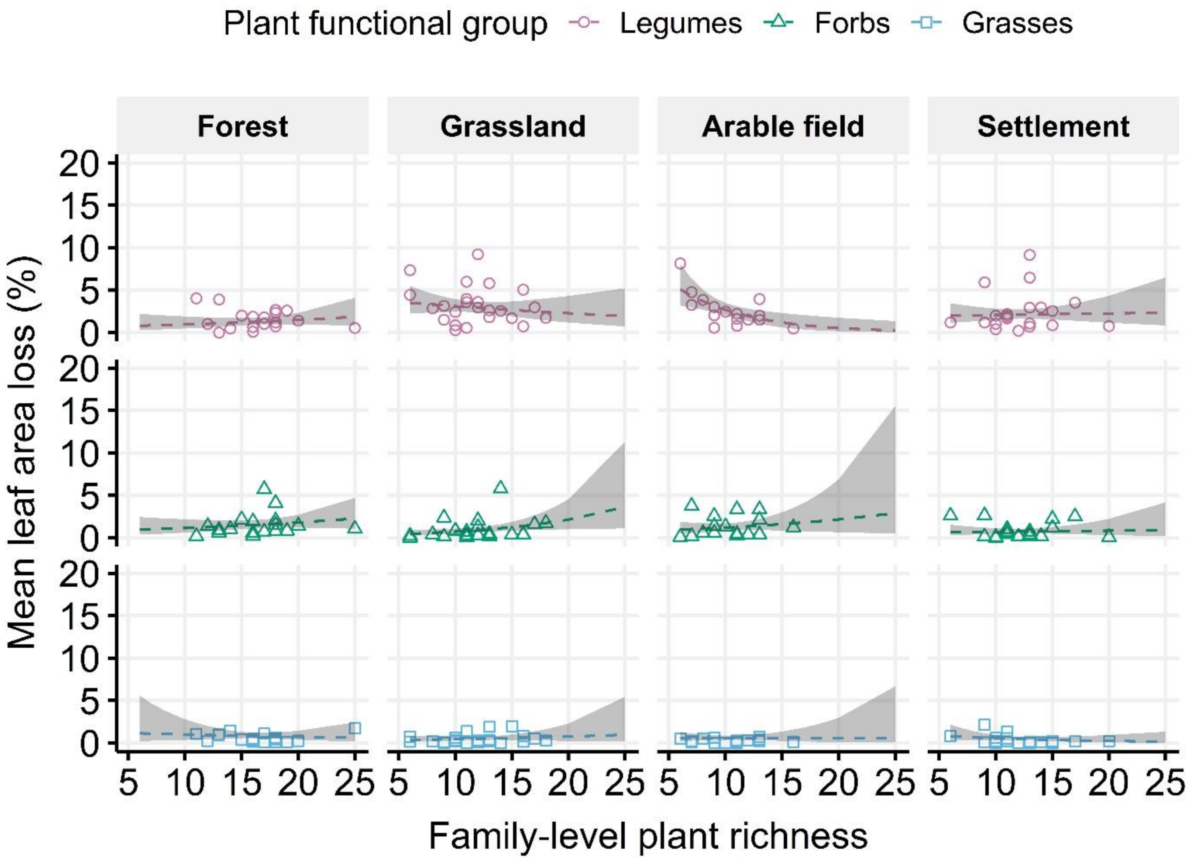

Fig. S5 Interactive effects of plant richness at family-level and habitat type with plant functional group (legumes: pink circles, non-leguminous forbs: green triangles: grasses: blue squares) on plot-averaged leaf area loss to leaf-chewing invertebrates. Lines indicate predictions of the full beta mixed model based on the complete data set (80 plots). Grey shades indicate 95% confidence bands. Three-way-interaction with habitat type was not supported. Model selection was done using ΔAICc and parsimony.

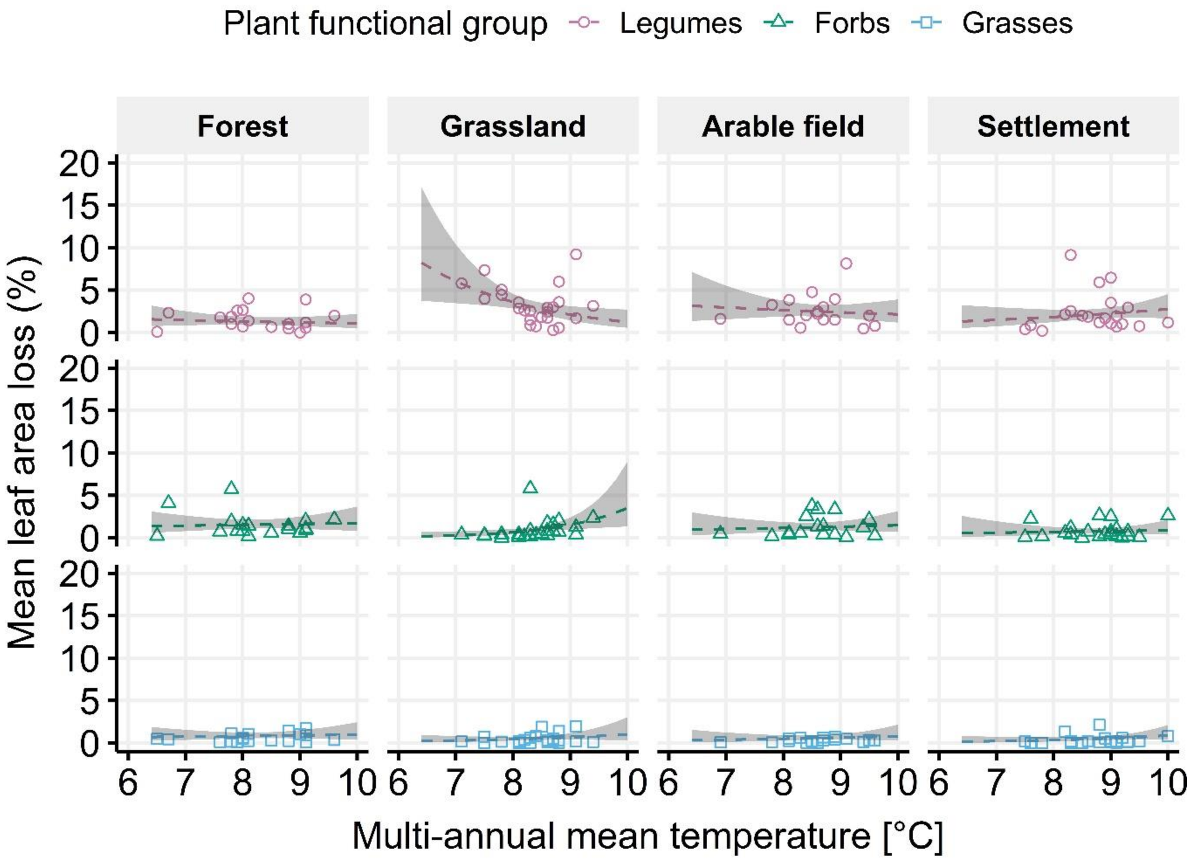

Fig. S6 Interactive effects of multi-annual mean temperature and habitat type with plant functional group (legumes: pink circles, non-leguminous forbs: green triangles: grasses: blue squares) on plot-averaged leaf area loss to leaf-chewing invertebrates. Lines indicate predictions of the full beta mixed model based on the complete data set (80 plots). Grey shades indicate 95% confidence bands. Interactions of herbivory on plant functional groups with multi-annual mean temperature is only supported in grassland plots (24 plots). Model selection was done using ΔAICc and parsimony.

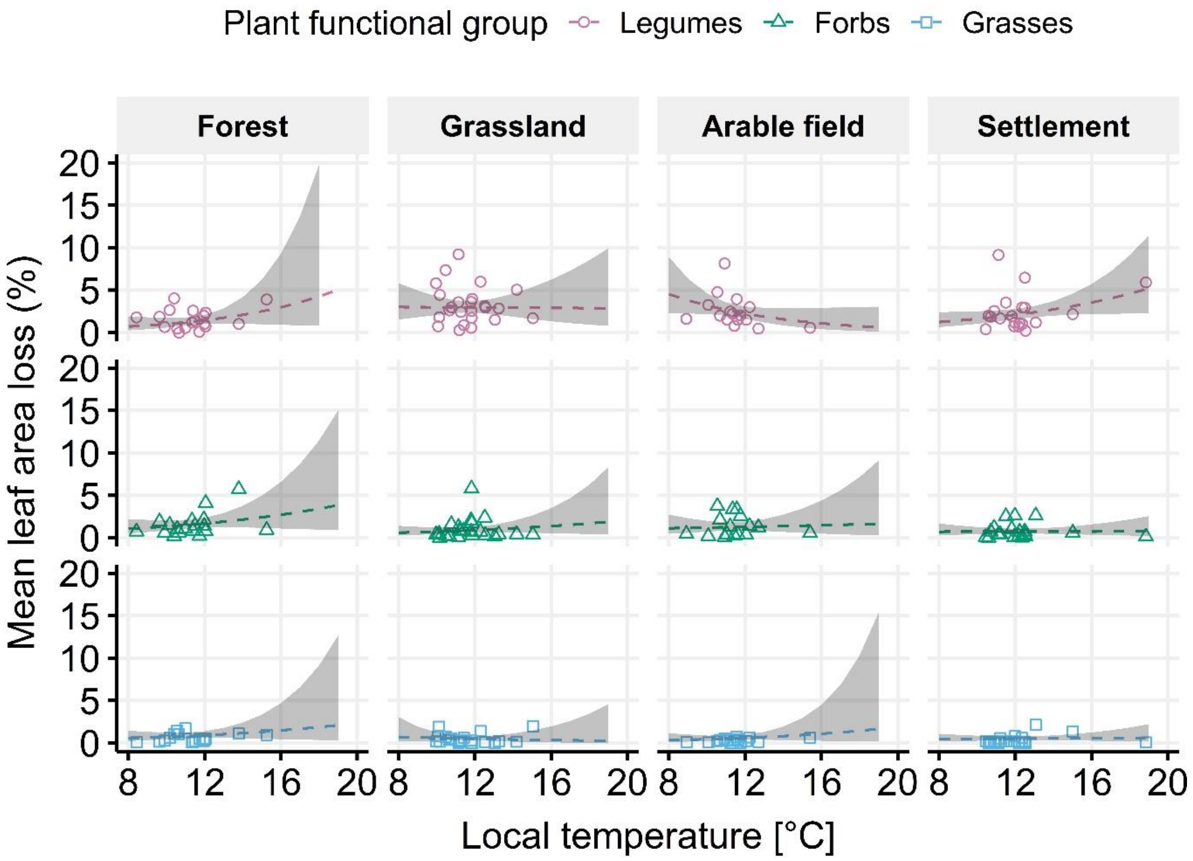

Fig. S7 Interactive effects of local mean temperature and habitat type with plant functional group (legumes: pink circles, non-leguminous forbs: green triangles: grasses: blue squares) on plot-averaged leaf area loss to leaf-chewing invertebrates. Lines indicate predictions of the full beta mixed model based on the complete data set (78 plots). Grey shades indicate 95% confidence bands. Interactions with local mean temperature were not supported, also not in the grassland subset (24 plots). Model selection was done using ΔAICc and parsimony.

Table S1 + Text: Dominant invertebrate feeding guilds among plant functional groups and leaves of different age

The invertebrate feeding guild dominating leaf damage was determined for 6460 leaves on 138 plots under consideration of leaf position (estimate of leaf age) and plant functional group (**Table S1**). Chewing and mining damage, but not sucking damage, was more frequently observed on basal leaves than apical leaves, thus older than younger leaves, across plant functional groups. Among plant functional groups, chewing folivory dominated, but differed in frequency of occurrence; 71% of leaves from legumes were dominated by chewing folivory, whereas only 29% and 26% of leaves from non-leguminous forbs and grasses, respectively. In turn, more leaves without damage were recorded in the group 'grasses' (66.2%) and 'forbs' (58.5%) than 'legumes' (26.4%). Mining and sucking damage as the dominant cause of leaf damage occurred rarely (< 7%), but more frequently on forbs (mining and sucking) and grasses (sucking) than on legumes (**Table S1**).

Invertebrate chewing folivory was quantified and present (> 0%) on 4029 leaves on 166 plots (info on leaf position and dominant invertebrate feeding guild incomplete). Leaf area loss to chewing folivory on an individual leaf, when present, commonly fell below 4%, but was higher on legumes (median: 1.3%, mean: 3.4%, 3rd quantile: 3.6%, max: 82.9%) than forbs (median: 0.9%, mean: 3.1%, 3rd quantile: 3.0%, max: 59.3%) and grasses (median: 0.4%, mean: 1.4%, 3rd quantile: 1.3%, max: 36.1%).

Invertebrate chewing folivory data on all three plant functional groups (minimum 10 leaves per functional group and plot) were obtained for 80 plots, as not all plant functional groups were represented on each plot. These data were used as plot-averaged proportional leaf area loss values to address questions on differential effects of temperature, land use and plant richness on invertebrate chewing folivory – the dominant leaf damage type – among plant functional groups.

Table S1 Overview on dominant leaf damage (based on damaged leaf area) of leaves (number, percent) per plant functional group and leaf position (A: apical/top/young, M: middle/intermediate-old, B: basal/old) by invertebrate feeding guild (chewer, sucker, miner, unknown, none).

	**Plant functional group**	**Legumes**				**Forbs**				**Grasses**			
		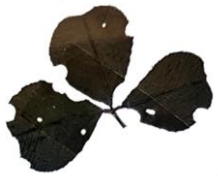				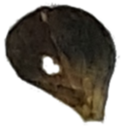				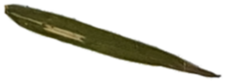			
**Dominant invertebrate feeding guild**	Leaf position	A	M	B	Σ	A	M	B	Σ	A	M	B	Σ
	Unit												
**Chewer**	-	333	369	374	1076	117	263	340	720	165	229	252	646
	%	63.5	74.2	74.8	70.7	14.3	32.3	41.6	29.4	19.6	27.9	30.6	26.0
**Sucker**	-	17	14	12	43	39	62	55	156	36	50	47	133
	%	3.2	2.8	2.4	2.8	4.8	7.6	6.7	6.4	4.3	6.1	5.7	5.3
**Miner**	-	0	2	2	4	15	35	41	91	2	3	7	12
	%	0.0	0.4	0.4	0.3	1.8	4.3	5.0	3.7	0.2	0.4	0.8	0.5
**Unknown**	-	0	2	2	4	8	9	5	22	2	11	16	29
	%	0.0	0.4	0.4	0.3	1.0	1.1	0.6	0.8	0.2	1.3	1.9	1.2
**None (no damage)**	-	174	110	110	394	642	445	376	1463	635	529	503	1667
	%	33.2	22.1	22.0	25.9	76.0	54.7	46.0	59.7	75.6	64.4	61.0	67.0
**Σ**	-	524	497	500	1521	821	814	817	2452	840	822	825	2487

Table S2 List of recorded plant species in vegetation survey and number of plots on which they occurred. Plant species list was used to derive plant richness measures (species and family level). Even though ferns, horsetails and woody plants are listed as part of the herb layer, they were not considered for herbivory assessment. No plant species occurred on all plots (= 179 plots).

Plant functional group	Family	Species	Number of plots
Forbs	Asteraceae	*Taraxacum* sect. *Ruderalia*	96
Forbs	Rubiaceae	*Galium album*	96
Forbs	Plantaginaceae	*Plantago lanceolata*	93
Forbs	Asteraceae	*Achillea millefolium* agg.	69
Forbs	Plantaginaceae	*Veronica chamaedrys*	65
Forbs	Ranunculaceae	*Ranunculus acris*	65
Forbs	Caryophyllaceae	*Cerastium holosteoides*	64
Forbs	Polygonaceae	*Rumex acetosa*	57
Forbs	Ranunculaceae	*Ranunculus repens*	56
Forbs	Urticaceae	*Urtica dioica*	50
Forbs	Lamiaceae	*Glechoma hederacea*	49
Forbs	Rosaceae	*Rubus* sect. *Rubus*	45
Forbs	Asteraceae	*Cirsium arvense*	43
Forbs	Rosaceae	*Potentilla reptans*	42
Forbs	Apiaceae	*Heracleum sphondylium*	39
Forbs	Convolvulaceae	*Convolvulus arvensis*	38
Forbs	Lamiaceae	*Ajuga reptans*	37
Forbs	Rubiaceae	*Galium aparine*	37
Forbs	Clusiaceae	*Hypericum perforatum*	36
Forbs	Rosaceae	*Rubus idaeus*	35
Forbs	Rosaceae	*Alchemilla vulgaris* agg.	34
Forbs	Caryophyllaceae	*Stellaria graminea*	33
Forbs	Lamiaceae	*Prunella vulgaris*	29
Forbs	Apiaceae	*Daucus carota*	28
Forbs	Primulaceae	*Lysimachia nummularia*	28
Forbs	Rosaceae	*Fragaria vesca*	28
Forbs	Rosaceae	*Geum urbanum*	26
Forbs	Lamiaceae	*Galeopsis tetrahit* agg.	25
Forbs	Asteraceae	*Crepis biennis*	23
Forbs	Polygonaceae	*Rumex obtusifolius*	23
Forbs	Juncaceae	*Juncus effusus*	22
Forbs	Asteraceae	*Leontodon hispidus*	21
Forbs	Asteraceae	*Centaurea jacea*	20
Forbs	Asteraceae	*Leucanthemum vulgare* agg.	20
Forbs	Campanulaceae	*Campanula patula*	18
Forbs	Dipsacaceae	*Knautia arvensis*	18
Forbs	Boraginaceae	*Myosotis arvensis*	17
Forbs	Plantaginaceae	*Veronica arvensis*	17
Forbs	Rosaceae	*Agrimonia eupatoria*	16
Forbs	Asteraceae	*Bellis perennis*	15

Forbs	Asteraceae	*Leontodon autumnalis*	15
Forbs	Rosaceae	*Sanguisorba minor*	15
Forbs	Rosaceae	*Sanguisorba officinalis*	15
Forbs	Apiaceae	*Aegopodium podagraria*	14
Forbs	Equisetaceae	*Equisetum arvense*	14
Forbs	Oxalidaceae	*Oxalis acetosella*	14
Forbs	Plantaginaceae	*Plantago major*	14
Forbs	Rubiaceae	*Galium verum*	14
Forbs	Asteraceae	*Hypochaeris radicata*	13
Forbs	Geraniaceae	*Geranium dissectum*	13
Forbs	Geraniaceae	*Geranium pratense*	13
Forbs	Apiaceae	*Pimpinella major*	12
Forbs	Juncaceae	*Luzula campestris*	12
Forbs	Onagraceae	*Epilobium angustifolium*	12
Forbs	Plantaginaceae	*Veronica officinalis*	12
Forbs	Scrophulariaceae	*Scrophularia nodosa*	12
Forbs	Violaceae	*Viola silvatica* agg.	12
Forbs	Asteraceae	*Tragopogon pratensis*	11
Forbs	Euphorbiaceae	*Euphorbia cyparissias*	11
Forbs	Orobanchaceae	*Rhinanthus minor*	11
Forbs	Plantaginaceae	*Plantago media*	11
Forbs	Polygonaceae	*Rumex crispus*	11
Forbs	Rosaceae	*Potentilla erecta*	11
Forbs	Asteraceae	*Tanacetum vulgare*	10
Forbs	Plantaginaceae	*Veronica persica*	10
Forbs	Valerianaceae	*Valerianella locusta*	10
Forbs	Caryophyllaceae	*Silene flos-cuculi*	9
Forbs	Dryopteridaceae	*Dryopteris carthusiana*	9
Forbs	Juncaceae	*Luzula multiflora*	9
Forbs	Lamiaceae	*Salvia pratensis*	9
Forbs	Violaceae	*Viola hirta*	9
Forbs	Asteraceae	*Cirsium palustre*	8
Forbs	Asteraceae	*Picris hieracioides*	8
Forbs	Asteraceae	*Tripleurospermum perforatum*	8
Forbs	Brassicaceae	*Capsella bursa-pastoris*	8
Forbs	Caryophyllaceae	*Stellaria holostea*	8
Forbs	Geraniaceae	*Geranium robertianum*	8
Forbs	Rosaceae	*Potentilla anserina*	8
Forbs	Asteraceae	*Erigeron annuus*	7
Forbs	Campanulaceae	*Campanula rotundifolia*	7
Forbs	Caryophyllaceae	*Moehringia trinervia*	7
Forbs	Caryophyllaceae	*Silene latifolia*	7
Forbs	Caryophyllaceae	*Stellaria media*	7
Forbs	Clusiaceae	*Hypericum maculatum*	7
Forbs	Dryopteridaceae	*Athyrium filix-femina*	7

Forbs	Lamiaceae	*Stachys sylvatica*	7
Forbs	Ranunculaceae	*Anemone nemorosa*	7
Forbs	Apiaceae	*Pimpinella saxifraga*	6
Forbs	Asteraceae	*Lactuca serriola*	6
Forbs	Asteraceae	*Lapsana communis*	6
Forbs	Balsaminaceae	*Impatiens parviflora*	6
Forbs	Brassicaceae	*Cardamine pratensis*	6
Forbs	Chenopodiaceae	*Chenopodium album*	6
Forbs	Colchicaceae	*Colchicum autumnale*	6
Forbs	-	Unidentified vascular plant species	6
Forbs	Geraniaceae	*Geranium pusillum*	6
Forbs	Lamiaceae	*Origanum vulgare*	6
Forbs	Plantaginaceae	*Veronica filiformis*	6
Forbs	Asteraceae	*Artemisia vulgaris*	5
Forbs	Balsaminaceae	*Impatiens glandulifera*	5
Forbs	Balsaminaceae	*Impatiens noli-tangere*	5
Forbs	Brassicaceae	*Thlaspi arvense*	5
Forbs	Caryophyllaceae	*Arenaria serpyllifolia*	5
Forbs	Caryophyllaceae	*Silene vulgaris*	5
Forbs	Juncaceae	*Juncus conglomeratus*	5
Forbs	Plantaginaceae	*Veronica serpyllifolia*	5
Forbs	Primulaceae	*Lysimachia nemorum*	5
Forbs	Saxifragaceae	*Saxifraga granulata*	5
Forbs	Violaceae	*Viola reichenbachiana*	5
Forbs	Apiaceae	*Chaerophyllum hirsutum*	4
Forbs	Asteraceae	*Cirsium oleraceum*	4
Forbs	Asteraceae	*Eupatorium cannabinum*	4
Forbs	Asteraceae	*Hieracium pilosella*	4
Forbs	Asteraceae	*Matricaria recutita*	4
Forbs	Asteraceae	*Solidago canadensis*	4
Forbs	Brassicaceae	*Alliaria petiolata*	4
Forbs	Convallariaceae	*Convallaria majalis*	4
Forbs	Geraniaceae	*Geranium molle*	4
Forbs	Lamiaceae	*Lamium purpureum*	4
Forbs	Lamiaceae	*Lycopus europaeus*	4
Forbs	Lamiaceae	*Thymus pulegioides*	4
Forbs	Onagraceae	*Circaea lutetiana*	4
Forbs	Onagraceae	*Epilobium* sp.	4
Forbs	Onagraceae	*Epilobium tetragonum*	4
Forbs	Plantaginaceae	*Linaria vulgaris*	4
Forbs	Polygonaceae	*Bistorta officinalis*	4
Forbs	Polygonaceae	*Polygonum aviculare*	4
Forbs	Polygonaceae	*Rumex acetosella*	4
Forbs	Ranunculaceae	*Ranunculus auricomus* agg.	4
Forbs	Rosaceae	*Filipendula ulmaria*	4

Forbs	Violaceae	*Viola arvensis*	4
Forbs	Asteraceae	*Arctium lappa*	3
Forbs	Asteraceae	*Cirsium vulgare*	3
Forbs	Asteraceae	*Tussilago farfara*	3
Forbs	Caryophyllaceae	*Cerastium glomeratum*	3
Forbs	Crassulaceae	*Sedum telephium*	3
Forbs	Dipsacaceae	*Knautia dipsacifolia*	3
Forbs	Dryopteridaceae	*Dryopteris dilatata*	3
Forbs	Equisetaceae	*Equisetum sylvaticum*	3
Forbs	Geraniaceae	*Geranium pyrenaicum*	3
Forbs	Juncaceae	*Luzula luzuloides*	3
Forbs	Lamiaceae	*Clinopodium vulgare*	3
Forbs	Plantaginaceae	*Veronica teucrium*	3
Forbs	Polygonaceae	*Rumex* sp.	3
Forbs	Polygonaceae	*Rumex conglomeratus*	3
Forbs	Primulaceae	*Lysimachia vulgaris*	3
Forbs	Primulaceae	*Primula veris*	3
Forbs	Ranunculaceae	*Ranunculus polyanthemos* agg.	3
Forbs	Rosaceae	*Potentilla argentea*	3
Forbs	Rubiaceae	*Galium boreale*	3
Forbs	Violaceae	*Viola canina*	3
Forbs	Alliaceae	*Allium* sp.	2
Forbs	Apiaceae	*Angelica sylvestris*	2
Forbs	Apiaceae	*Anthriscus sylvestris*	2
Forbs	Apiaceae	*Carum carvi*	2
Forbs	Apiaceae	*Chaerophyllum bulbosum*	2
Forbs	Apiaceae	*Falcaria vulgaris*	2
Forbs	Apiaceae	*Pastinaca sativa*	2
Forbs	Apiaceae	*Seseli libanotis*	2
Forbs	Apiaceae	*Silaum silaus*	2
Forbs	Asteraceae	*Aposeris foetida*	2
Forbs	Asteraceae	*Centaurea scabiosa*	2
Forbs	Asteraceae	*Cichorium intybus*	2
Forbs	Asteraceae	*Hieracium aurantiacum*	2
Forbs	Asteraceae	*Hieracium piloselloides*	2
Forbs	Asteraceae	*Hieracium sabaudum*	2
Forbs	Asteraceae	*Prenanthes purpurea*	2
Forbs	Asteraceae	*Senecio ovatus*	2
Forbs	Asteraceae	*Senecio sylvaticus*	2
Forbs	Asteraceae	*Senecio vulgaris*	2
Forbs	Boraginaceae	*Symphytum officinale*	2
Forbs	Brassicaceae	*Barbarea vulgaris*	2
Forbs	Campanulaceae	*Phyteuma orbiculare*	2
Forbs	Campanulaceae	*Phyteuma spicatum*	2
Forbs	Caryophyllaceae	Unidentified species	2

Forbs	Caryophyllaceae	*Cerastium arvense*	2
Forbs	Caryophyllaceae	*Stellaria alsine*	2
Forbs	Caryophyllaceae	*Stellaria aquatica*	2
Forbs	Clusiaceae	*Hypericum pulchrum*	2
Forbs	Clusiaceae	*Hypericum tetrapterum*	2
Forbs	Convallariaceae	*Polygonatum verticillatum*	2
Forbs	Convolvulaceae	*Calystegia sepium*	2
Forbs	Dipsacaceae	*Dipsacus fullonum*	2
Forbs	Dipsacaceae	*Scabiosa columbaria*	2
Forbs	Euphorbiaceae	*Mercurialis perennis*	2
Forbs	Juncaceae	*Luzula sylvatica*	2
Forbs	Lamiaceae	*Betonica officinalis*	2
Forbs	Lamiaceae	*Lamium galeobdolon* agg.	2
Forbs	Lamiaceae	*Lamium maculatum*	2
Forbs	Lamiaceae	*Mentha longifolia*	2
Forbs	Lamiaceae	*Salvia glutinosa*	2
Forbs	Lythraceae	*Lythrum salicaria*	2
Forbs	Malvaceae	*Malva moschata*	2
Forbs	Onagraceae	*Epilobium hirsutum*	2
Forbs	Onagraceae	*Epilobium tetragonum* ssp. *lamyi*	2
Forbs	Orobanchaceae	*Rhinanthus alectorolophus*	2
Forbs	Papaveraceae	*Papaver rhoeas*	2
Forbs	Plantaginaceae	*Veronica montana*	2
Forbs	Polygonaceae	*Persicaria amphibia*	2
Forbs	Polygonaceae	Unidentified species	2
Forbs	Ranunculaceae	*Clematis vitalba*	2
Forbs	Ranunculaceae	*Ranunculus ficaria*	2
Forbs	Ranunculaceae	*Ranunculus lanuginosus*	2
Forbs	Rosaceae	*Potentilla* sp.	2
Forbs	Rubiaceae	*Galium saxatile*	2
Forbs	Scrophulariaceae	*Verbascum nigrum*	2
Forbs	Solanaceae	*Atropa bella-donna*	2
Forbs	Valerianaceae	*Valeriana officinalis*	2
Forbs	Violaceae	*Viola* sp.	2
Forbs	Violaceae	*Viola odorata*	2
Forbs	Alliaceae	*Allium carinatum*	1
Forbs	Alliaceae	*Allium schoenoprasum*	1
Forbs	Anthericaceae	*Anthericum ramosum*	1
Forbs	Apiaceae	*Astrantia major*	1
Forbs	Apiaceae	*Bupleurum falcatum*	1
Forbs	Apiaceae	*Chaerophyllum aureum*	1
Forbs	Apiaceae	*Chaerophyllum temulum*	1
Forbs	Apiaceae	*Eryngium campestre*	1
Forbs	Apiaceae	*Sanicula europaea*	1
Forbs	Apiaceae	*Torilis arvensis*	1

Forbs	Araliaceae	*Hedera helix*	1
Forbs	Asparagaceae	*Asparagus officinalis*	1
Forbs	Asteraceae	*Anthemis tinctoria*	1
Forbs	Asteraceae	*Arctium* sp.	1
Forbs	Asteraceae	Unidentified species	1
Forbs	Asteraceae	*Buphthalmum salicifolium*	1
Forbs	Asteraceae	*Centaurea cyanus*	1
Forbs	Asteraceae	*Cichorium endivia*	1
Forbs	Asteraceae	*Cirsium* sp.	1
Forbs	Asteraceae	*Cirsium eriophorum*	1
Forbs	Asteraceae	*Cirsium heterophyllum*	1
Forbs	Asteraceae	*Crepis* sp.	1
Forbs	Asteraceae	*Hieracium* sp.	1
Forbs	Asteraceae	*Matricaria discoidea*	1
Forbs	Asteraceae	*Mycelis muralis*	1
Forbs	Asteraceae	*Petasites albus*	1
Forbs	Asteraceae	*Solidago virgaurea*	1
Forbs	Asteraceae	*Sonchus asper*	1
Forbs	Asteraceae	*Sonchus oleraceus*	1
Forbs	Boraginaceae	*Myosotis* sp.	1
Forbs	Boraginaceae	*Myosotis scorpioides*	1
Forbs	Boraginaceae	*Myosotis sylvatica*	1
Forbs	Brassicaceae	*Arabis glabra*	1
Forbs	Brassicaceae	*Brassica napus*	1
Forbs	Brassicaceae	*Brassicaceae*	1
Forbs	Brassicaceae	*Bunias orientalis*	1
Forbs	Brassicaceae	*Cardaria draba*	1
Forbs	Brassicaceae	*Hesperis matronalis*	1
Forbs	Brassicaceae	*Lepidium campestre*	1
Forbs	Brassicaceae	*Rorippa austriaca*	1
Forbs	Brassicaceae	*Sisymbrium officinale*	1
Forbs	Campanulaceae	*Campanula* sp.	1
Forbs	Campanulaceae	*Campanula persicifolia*	1
Forbs	Campanulaceae	*Campanula trachelium*	1
Forbs	Caryophyllaceae	*Cerastium* sp.	1
Forbs	Caryophyllaceae	*Cerastium lucorum*	1
Forbs	Caryophyllaceae	*Dianthus deltoides*	1
Forbs	Caryophyllaceae	*Herniaria glabra*	1
Forbs	Caryophyllaceae	*Silene dioica*	1
Forbs	Caryophyllaceae	*Silene nutans*	1
Forbs	Caryophyllaceae	*Stellaria nemorum*	1
Forbs	Clusiaceae	*Hypericum hirsutum*	1
Forbs	Crassulaceae	*Sedum acre*	1
Forbs	Dennstaedtiaceae	*Pteridium aquilinum*	1
Forbs	Dipsacaceae	*Knautia* sp.	1

Forbs	Dipsacaceae	*Scabiosa columbaria* agg.	1
Forbs	Dipsacaceae	*Succisa pratensis*	1
Forbs	Dryopteridaceae	*Dryopteris expansa*	1
Forbs	Dryopteridaceae	*Dryopteris filix-mas*	1
Forbs	Equisetaceae	*Equisetum palustre*	1
Forbs	Euphorbiaceae	*Euphorbia amygdaloides*	1
Forbs	Euphorbiaceae	*Euphorbia seguieriana*	1
Forbs	Euphorbiaceae	*Mercurialis ovata*	1
Forbs	Gentianaceae	*Centaurium erythraea*	1
Forbs	Geraniaceae	*Geranium columbinum*	1
Forbs	Lamiaceae	*Acinos alpinus*	1
Forbs	Lamiaceae	*Ajuga genevensis*	1
Forbs	Lamiaceae	*Galeopsis speciosa*	1
Forbs	Lamiaceae	Unidentified species	1
Forbs	Lamiaceae	*Lamium album*	1
Forbs	Lamiaceae	*Lamium montanum*	1
Forbs	Lamiaceae	*Mentha arvensis*	1
Forbs	Lamiaceae	*Mentha spicata* agg.	1
Forbs	Lamiaceae	*Scutellaria galericulata*	1
Forbs	Lamiaceae	*Stachys palustris*	1
Forbs	Lamiaceae	*Teucrium chamaedrys*	1
Forbs	Lamiaceae	*Teucrium scorodonia*	1
Forbs	Lamiaceae	*Thymus praecox*	1
Forbs	Linaceae	*Linum catharticum*	1
Forbs	Onagraceae	*Epilobium collinum*	1
Forbs	Onagraceae	*Epilobium montanum*	1
Forbs	Onagraceae	*Epilobium obscurum*	1
Forbs	Onagraceae	*Epilobium palustre*	1
Forbs	Onagraceae	*Epilobium parviflorum*	1
Forbs	Orchidaceae	*Cephalanthera rubra*	1
Forbs	Orobanchaceae	*Euphrasia* sp.	1
Forbs	Orobanchaceae	*Melampyrum arvense*	1
Forbs	Orobanchaceae	*Melampyrum pratense*	1
Forbs	Orobanchaceae	*Melampyrum sylvaticum*	1
Forbs	Orobanchaceae	*Orobanche gracilis*	1
Forbs	Oxalidaceae	*Oxalis stricta*	1
Forbs	Papaveraceae	*Chelidonium majus*	1
Forbs	Papaveraceae	*Papaver dubium*	1
Forbs	Plantaginaceae	*Chaenorhinum minus*	1
Forbs	Plantaginaceae	*Digitalis purpurea*	1
Forbs	Plantaginaceae	*Veronica agrestis*	1
Forbs	Plantaginaceae	*Veronica hederifolia*	1
Forbs	Polygalaceae	*Polygala chamaebuxus*	1
Forbs	Polygalaceae	*Polygala comosa*	1
Forbs	Polygonaceae	*Persicaria* sp.	1

Forbs	Polygonaceae	*Persicaria maculosa*	1
Forbs	Polygonaceae	*Polygonum* sp.	1
Forbs	Primulaceae	*Lysimachia punctata*	1
Forbs	Primulaceae	*Primula* sp.	1
Forbs	Primulaceae	*Primula elatior*	1
Forbs	Ranunculaceae	*Aquilegia vulgaris*	1
Forbs	Rosaceae	*Alchemilla mollis*	1
Forbs	Rosaceae	*Geum rivale*	1
Forbs	Rubiaceae	*Cruciata laevipes*	1
Forbs	Rubiaceae	*Galium odoratum*	1
Forbs	Rubiaceae	*Galium pumilum*	1
Forbs	Rubiaceae	*Galium rotundifolium*	1
Forbs	Rubiaceae	*Galium uliginosum*	1
Forbs	Rubiaceae	*Sherardia arvensis*	1
Forbs	Scrophulariaceae	*Verbascum* sp.	1
Forbs	Thelypteridaceae	*Oreopteris limbosperma*	1
Forbs	Thelypteridaceae	*Phegopteris connectilis*	1
Forbs	Trilliaceae	*Paris quadrifolia*	1
Forbs	Valerianaceae	*Valeriana officinalis* agg.	1
Forbs	Violaceae	*Viola mirabilis*	1
Forbs	Violaceae	*Viola riviniana*	1
Grasses	Poaceae	*Dactylis glomerata*	129
Grasses	Poaceae	*Poa pratensis*	98
Grasses	Poaceae	*Arrhenatherum elatius*	95
Grasses	Poaceae	*Poa trivialis*	84
Grasses	Poaceae	*Alopecurus pratensis*	69
Grasses	Poaceae	*Elymus repens*	68
Grasses	Poaceae	*Festuca pratensis*	65
Grasses	Poaceae	*Festuca rubra* agg.	65
Grasses	Poaceae	*Lolium perenne*	59
Grasses	Poaceae	*Holcus lanatus*	58
Grasses	Poaceae	*Phleum pratense*	52
Grasses	Poaceae	*Trisetum flavescens*	50
Grasses	Poaceae	*Agrostis capillaris*	48
Grasses	Poaceae	*Anthoxanthum odoratum*	35
Grasses	Cyperaceae	*Carex hirta*	25
Grasses	Poaceae	*Agrostis gigantea*	22
Grasses	Poaceae	*Cynosurus cristatus*	19
Grasses	Cyperaceae	*Carex muricata* agg.	18
Grasses	Poaceae	*Calamagrostis epigejos*	18
Grasses	Poaceae	*Deschampsia cespitosa*	18
Grasses	Cyperaceae	*Carex brizoides*	17
Grasses	Poaceae	*Bromus hordeaceus*	17
Grasses	Cyperaceae	*Carex sylvatica*	15
Grasses	Poaceae	*Holcus mollis*	15

Grasses	Cyperaceae	*Carex flacca*	14
Grasses	Poaceae	*Brachypodium sylvaticum*	14
Grasses	Cyperaceae	*Carex ovalis*	13
Grasses	Poaceae	*Poa angustifolia*	12
Grasses	Poaceae	*Bromus sterilis*	11
Grasses	Poaceae	*Lolium multiflorum*	11
Grasses	Poaceae	*Brachypodium pinnatum*	10
Grasses	Poaceae	*Festuca ovina* agg.	10
Grasses	Poaceae	*Helictotrichon pubescens*	9
Grasses	Poaceae	*Triticum aestivum*	9
Grasses	Cyperaceae	*Carex pallescens*	8
Grasses	Cyperaceae	*Carex remota*	7
Grasses	Poaceae	*Brachypodium rupestre*	7
Grasses	Poaceae	*Bromus erectus*	7
Grasses	Poaceae	*Poa nemoralis*	7
Grasses	Poaceae	*Briza media*	6
Grasses	Poaceae	*Bromus inermis*	6
Grasses	Poaceae	*Poa annua*	6
Grasses	Cyperaceae	*Carex pilulifera*	5
Grasses	Poaceae	*Deschampsia flexuosa*	5
Grasses	Poaceae	*Danthonia decumbens*	4
Grasses	Poaceae	*Festuca arundinacea*	4
Grasses	Poaceae	*Phragmites australis*	4
Grasses	Cyperaceae	*Carex flava*	3
Grasses	Poaceae	*Alopecurus myosuroides*	3
Grasses	Poaceae	*Elymus caninus*	3
Grasses	Poaceae	*Festuca* sp.	3
Grasses	Poaceae	*Melica uniflora*	3
Grasses	Poaceae	*Milium effusum*	3
Grasses	Poaceae	*Phalaris arundinacea*	3
Grasses	Poaceae	*Poa* sp.	3
Grasses	Cyperaceae	*Carex* sp.	2
Grasses	Cyperaceae	*Carex caryophyllea*	2
Grasses	Cyperaceae	*Carex montana*	2
Grasses	Cyperaceae	*Scirpus sylvaticus*	2
Grasses	Poaceae	*Agrostis stolonifera* agg.	2
Grasses	Poaceae	*Calamagrostis arundinacea*	2
Grasses	Poaceae	*Calamagrostis varia*	2
Grasses	Poaceae	*Festuca gigantea*	2
Grasses	Poaceae	*Hordeum vulgare*	2
Grasses	Poaceae	*Molinia caerulea*	2
Grasses	Poaceae	*Secale cereale*	2
Grasses	Poaceae	*Sesleria albicans*	2
Grasses	Cyperaceae	*Carex alba*	1
Grasses	Cyperaceae	*Carex digitata*	1

Grasses	Cyperaceae	*Carex humilis*	1
Grasses	Cyperaceae	*Carex nigra*	1
Grasses	Cyperaceae	*Carex panicea*	1
Grasses	Cyperaceae	*Carex vulpina*	1
Grasses	Poaceae	*Apera spica-venti*	1
Grasses	Poaceae	*Bromus arvensis*	1
Grasses	Poaceae	*Bromus benekenii*	1
Grasses	Poaceae	*Bromus secalinus*	1
Grasses	Poaceae	*Festuca heterophylla*	1
Grasses	Poaceae	*Glyceria notata*	1
Grasses	Poaceae	*Hordelymus europaeus*	1
Grasses	Poaceae	*Koeleria pyramidata*	1
Grasses	Poaceae	*Melica nutans*	1
Grasses	Poaceae	*Molinia arundinacea*	1
Grasses	Poaceae	Unidentified species	1
Grasses	Poaceae	*Stipa pennata*	1
Legumes	Fabaceae	*Trifolium repens*	76
Legumes	Fabaceae	*Trifolium pratense*	63
Legumes	Fabaceae	*Lotus corniculatus*	49
Legumes	Fabaceae	*Lathyrus pratensis*	48
Legumes	Fabaceae	*Vicia sepium*	44
Legumes	Fabaceae	*Vicia cracca*	32
Legumes	Fabaceae	*Trifolium dubium*	24
Legumes	Fabaceae	*Vicia hirsuta*	22
Legumes	Fabaceae	*Medicago lupulina*	20
Legumes	Fabaceae	*Vicia angustifolia*	17
Legumes	Fabaceae	*Trifolium medium*	9
Legumes	Fabaceae	*Vicia sativa*	9
Legumes	Fabaceae	*Vicia tetrasperma*	9
Legumes	Fabaceae	*Securigera varia*	6
Legumes	Fabaceae	*Lathyrus tuberosus*	5
Legumes	Fabaceae	*Medicago* x *varia*	5
Legumes	Fabaceae	*Anthyllis vulneraria*	4
Legumes	Fabaceae	*Medicago falcata*	3
Legumes	Fabaceae	*Melilotus officinalis*	3
Legumes	Fabaceae	*Onobrychis viciifolia*	3
Legumes	Fabaceae	*Astragalus glycyphyllos*	2
Legumes	Fabaceae	*Lathyrus linifolius*	2
Legumes	Fabaceae	*Lupinus polyphyllus*	2
Legumes	Fabaceae	*Ononis repens*	2
Legumes	Fabaceae	*Ononis spinosa* agg.	2
Legumes	Fabaceae	Unidentified species	1
Legumes	Fabaceae	*Hippocrepis comosa*	1
Legumes	Fabaceae	*Lathyrus sylvestris*	1
Legumes	Fabaceae	*Lathyrus vernus*	1

Legumes	Fabaceae	*Lotus pedunculatus*	1
Legumes	Fabaceae	*Medicago minima*	1
Legumes	Fabaceae	*Melilotus albus*	1
Legumes	Fabaceae	*Trifolium alpestre*	1
Legumes	Fabaceae	*Trifolium hybridum*	1
Legumes	Fabaceae	*Trifolium incarnatum*	1
Legumes	Fabaceae	*Vicia pannonica*	1
Woody plants	Fagaceae	*Quercus robur*	17
Woody plants	Rosaceae	*Prunus* sp.	17
Woody plants	Aceraceae	*Acer pseudoplatanus*	14
Woody plants	Cornaceae	*Cornus sanguinea*	13
Woody plants	Aceraceae	*Acer platanoides*	11
Woody plants	Pinaceae	*Picea abies*	11
Woody plants	Fagaceae	*Fagus sylvatica*	9
Woody plants	Oleaceae	*Fraxinus excelsior*	9
Woody plants	Rosaceae	*Crataegus monogyna*	9
Woody plants	Rosaceae	*Sorbus aucuparia*	9
Woody plants	Betulaceae	*Betula pendula*	8
Woody plants	Ericaceae	*Vaccinium myrtillus*	7
Woody plants	Aceraceae	*Acer campestre*	6
Woody plants	Pinaceae	*Pinus sylvestris*	6
Woody plants	Rosaceae	*Rosa* sp.	6
Woody plants	Betulaceae	*Carpinus betulus*	5
Woody plants	Rosaceae	*Rosa canina*	5
Woody plants	Salicaceae	*Salix caprea*	5
Woody plants	Caprifoliaceae	*Sambucus nigra*	4
Woody plants	Celastraceae	*Euonymus europaea*	4
Woody plants	Pinaceae	*Abies alba*	4
Woody plants	Rosaceae	*Prunus spinosa*	4
Woody plants	Salicaceae	*Populus nigra*	4
Woody plants	Salicaceae	*Salix* sp.	4
Woody plants	Betulaceae	*Corylus avellana*	3
Woody plants	Ericaceae	*Calluna vulgaris*	3
Woody plants	Aceraceae	*Acer* sp.	2
Woody plants	Caprifoliaceae	*Lonicera xylosteum*	2
Woody plants	Ericaceae	*Vaccinium vitis-idaea*	2
Woody plants	Fagaceae	*Quercus* sp.	2
Woody plants	Oleaceae	*Ligustrum vulgare*	2
Woody plants	Rhamnaceae	*Frangula alnus*	2
Woody plants	Salicaceae	*Populus tremula*	2
Woody plants	Berberidaceae	*Mahonia aquifolium*	1
Woody plants	Cornaceae	*Cornus sanguinea* ssp. *sanguinea*	1
Woody plants	Fagaceae	*Quercus petraea*	1
Woody plants	Fagaceae	*Quercus rubra*	1
Woody plants	Grossulariaceae	*Ribes rubrum*	1

Woody plants	Pinaceae	*Larix decidua*	1
Woody plants	Rosaceae	*Prunus avium*	1
Woody plants	Rosaceae	*Sorbus* sp.	1
Woody plants	Rosaceae	*Sorbus torminalis*	1

Table S3 Pearson correlation coefficients for all continuous variables included in model selection processes on herbivory data (80 plots, 78 plots when including local mean temperature). Significant correlations based on α = 0.05 are indicated as following: *, $P < 0.05$; **, $P < 0.01$; ***, $P < 0.001$

		Pearson correlation coefficients				
Scale [km]	Variable	Temp (1)	MAT (2)	specnum (3)	famnum (4)	Gra% (5)
-	Local mean temperature (1)					
-	Multi-annual mean temperature (2)	0.23***				
-	Species-level plant richness (3)	-0.03	-0.02			
-	Family-level plant richness (4)	-0.09	-0.05	0.76***		
0.2	Grassland percentage (5)	-0.04	-0.09	-0.15*	-0.23***	
0.5	Grassland percentage	0.10	-0.16*	-0.17*	-0.21***	
1.0	Grassland percentage	0.09	-0.29***	-0.14*	-0.14*	
1.5	Grassland percentage	0.07	-0.42***	-0.11	-0.13*	
2.0	Grassland percentage	0.06	-0.47***	-0.09	-0.11	
2.5	Grassland percentage	0.06	-0.51***	-0.07	-0.10	
3.0	Grassland percentage	0.07	-0.52***	-0.06	-0.09	
0.2	Landscape diversity	0.12	0.19**	-0.22***	-0.40***	0.39***
0.5	Landscape diversity	0.29***	0.13	-0.14*	-0.29***	0.44***
1.0	Landscape diversity	0.17**	0.05	-0.18**	-0.18**	0.51***
1.5	Landscape diversity	0.10	0.03	-0.18**	-0.19**	0.49***
2.0	Landscape diversity	0.07	0.00	-0.16*	-0.17**	0.44***
2.5	Landscape diversity	0.10	-0.00	-0.14*	-0.18**	0.39***
3.0	Landscape diversity	0.12	-0.04	-0.14*	-0.19**	0.37***

Table S4 Null, "full" and best beta mixed models on mean leaf area loss by chewing invertebrates per plant functional group and plot (80 plots). "Full" models include different sets of fixed effects but always include plant functional group, one climatic environmental variable, one land-use or plant-richness variable and their interaction terms. Fixed effects encompass: Plant functional group (Plant guild), multi-annual mean temperature (MAT), habitat type (habitat), species-level plant richness (specnum) and family-level plant richness (famnum), and proportion grassland (Gra) and landscape diversity (LD) at multiple spatial scales. Continuous predictor variables were z-transformed (s-Fixed effect) prior to modelling. To account for study design, plot nested in region was added as random term. Asterisks (*) between fixed effects indicate that both, all main effects and all interaction terms were included. Bold font indicates the best model based on relative goodness of model fit (lowest AICc).

Spatial scale [km]	Model	Fixed effects	Random effects	df	AICc
-	NULL	-	Region/Plot	4	-1606.3
-	"full"	Plant guild*sMAT*habitat	Region/Plot	27	-1686.6
	**best**	**Plant guild*habitat**	**Region/Plot**	**15**	**-1697.7**
-	"full"	Plant guild*sMAT*sspecnum	Region/Plot	15	-1685.3
-	best	Plant guild	Region/Plot	6	-1688.6
-	"full"	Plant guild*sMAT*sfamnum	Region/Plot	15	-1698.1
-	**best**	**Plant guild*sfamnum**	**Region/Plot**	**9**	**-1699.0**
0.2	"full"	Plant guild*sMAT*sGra	Region/Plot	15	-1684.5
0.2	best	Plant guild	Region/Plot	6	-1688.6
0.5	"full"	Plant guild*sMAT*sGra	Region/Plot	15	-1676.4
0.5	best	Plant guild	Region/Plot	6	-1688.6
1.0	"full"	Plant guild*sMAT*sGra	Region/Plot	15	-1673.6
1.0	best	Plant guild	Region/Plot	6	-1688.6
1.5	"full"	Plant guild*sMAT*sGra	Region/Plot	15	-1672.9
1.5	best	Plant guild	Region/Plot	6	-1688.6
2.0	"full"	Plant guild*sMAT*sGra	Region/Plot	15	-1673.2
2.0	best	Plant guild	Region/Plot	6	-1688.6
2.5	"full"	Plant guild*sMAT*sGra	Region/Plot	15	-1674.3
2.5	best	Plant guild	Region/Plot	6	-1688.6
3.0	"full"	Plant guild*sMAT*sGra	Region/Plot	15	-1675.3
3.0	best	Plant guild	Region/Plot	6	-1688.6
0.2	"full"	Plant guild*sMAT*sLD	Region/Plot	15	-1684.0
0.2	best	Plant guild	Region/Plot	6	-1688.6
0.5	"full"	Plant guild*sMAT*sLD	Region/Plot	15	-1675.6
0.5	best	Plant guild	Region/Plot	6	-1688.6
1.0	"full"	Plant guild*sMAT*sLD	Region/Plot	15	-1673.3
1.0	best	Plant guild	Region/Plot	6	-1688.6
1.5	"full"	Plant guild*sMAT*sLD	Region/Plot	15	-1673.6
1.5	best	Plant guild	Region/Plot	6	-1688.6
2.0	"full"	Plant guild*sMAT*sLD	Region/Plot	15	-1674.5
2.0	best	Plant guild	Region/Plot	6	-1688.6
2.5	"full"	Plant guild*sMAT*sLD	Region/Plot	15	-1675.9
2.5	best	Plant guild	Region/Plot	6	-1688.6
3.0	"full"	Plant guild*sMAT*sLD	Region/Plot	15	-1676.9
3.0	best	Plant guild	Region/Plot	6	-1688.6

Table S5 Null, "full" and best beta mixed models on mean leaf area loss by chewing invertebrates per plant functional group and plot (78 plots). "Full" models include different sets of fixed effects but always include plant functional group, one climatic environmental variable, one land-use or plant-richness variable and their interaction terms. Fixed effects encompass: Plant functional group (Plant guild), local mean temperature of the 1-month period prior to leaf sampling (Temp), habitat type (habitat), species-level plant richness (specnum) and family-level plant richness (famnum), and proportion grassland (Gra) and landscape diversity (LD) at multiple spatial scales. Continuous predictor variables were z-transformed (s-Fixed effect) prior to modelling. To account for study design, plot nested in region was added as random term. Asterisks (*) between fixed effects indicate that both, all main effects and all interaction terms were included. Bold font indicates the best model based on relative goodness of model fit (lowest AICc).

Spatial scale [km]	Model	Fixed effects	Random effects	df	AICc
-	NULL	-	Region/Plot	4	-1565.6
-	"full"	Plant guild*sTemp*habitat	Region/Plot	27	-1635.1
	**best**	**Plant guild*habitat**	**Region/Plot**	**15**	**-1653.4**
-	"full"	Plant guild*sTemp*sspecnum	Region/Plot	15	-1635.5
-	best	Plant guild	Region/Plot	6	-1644.2
-	"full"	Plant guild*sTemp*sfamnum	Region/Plot	15	-1641.7
-	**best**	**Plant guild*sfamnum**	**Region/Plot**	**9**	**-1653.5**
0.2	"full"	Plant guild*sTemp*sGra	Region/Plot	15	-1633.1
0.2	best	Plant guild	Region/Plot	6	-1644.2
0.5	"full"	Plant guild*sTemp*sGra	Region/Plot	15	-1630.3
0.5	best	Plant guild	Region/Plot	6	-1644.2
1.0	"full"	Plant guild*sTemp*sGra	Region/Plot	15	-1628.7
1.0	best	Plant guild	Region/Plot	6	-1644.2
1.5	"full"	Plant guild*sTemp*sGra	Region/Plot	15	-1630.3
1.5	best	Plant guild	Region/Plot	6	-1644.2
2.0	"full"	Plant guild*sTemp*sGra	Region/Plot	15	-1630.7
2.0	best	Plant guild	Region/Plot	6	-1644.2
2.5	"full"	Plant guild*sTemp*sGra	Region/Plot	15	-1630.9
2.5	best	Plant guild	Region/Plot	6	-1644.2
3.0	"full"	Plant guild*sTemp*sGra	Region/Plot	15	-1630.8
3.0	best	Plant guild	Region/Plot	6	-1644.2
0.2	"full"	Plant guild*sTemp*sLD	Region/Plot	15	-1630.9
0.2	best	Plant guild	Region/Plot	6	-1644.2
0.5	"full"	Plant guild*sTemp*sLD	Region/Plot	15	-1629.7
0.5	best	Plant guild	Region/Plot	6	-1644.2
1.0	"full"	Plant guild*sTemp*sLD	Region/Plot	15	-1627.7
1.0	best	Plant guild	Region/Plot	6	-1644.2
1.5	"full"	Plant guild*sTemp*sLD	Region/Plot	15	-1628.2
1.5	best	Plant guild	Region/Plot	6	-1644.2
2.0	"full"	Plant guild*sTemp*sLD	Region/Plot	15	-1628.9
2.0	best	Plant guild	Region/Plot	6	-1644.2
2.5	"full"	Plant guild*sTemp*sLD	Region/Plot	15	-1628.7
2.5	best	Plant guild	Region/Plot	6	-1644.2
3.0	"full"	Plant guild*sTemp*sLD	Region/Plot	15	-1629.1
3.0	best	Plant guild	Region/Plot	6	-1644.2

Table S6 Selection process of beta mixed models on plot-averaged leaf area loss by chewing invertebrates per plant functional group on data from grassland habitats only (24 plots). Fixed effects encompass plant functional group (Plant guild) and z-transformed multi-annual mean temperature (sMAT) or local mean temperature 1-month prior to leaf sampling (sTemp). As a single habitat type was subsetted, no random term was added. Asterisks (*) between fixed effects indicate that both, main effects and all interaction terms were included. Relative goodness of model fit is indicated by Akaike's information criterion corrected for small sample size (AICc). Bold font highlights the best model based on ΔAICc < 2.

Fixed effects	Random effects	df	AICc	ΔAICc
**Plant guild*sMAT**	**Plot**	**8**	**-511.6**	**0.00**
Plant guild+sMAT	Plot	6	-504.5	7.14
Plant guild	Plot	5	-506.6	5.05
sMAT	Plot	4	-468.4	43.20
- (Null model)	Plot	3	-468.1	43.48
Plant guild*sTemp	Plot	8	-500.2	6.41
Plant guild+sTemp	Plot	6	-504.3	2.27
**Plant guild**	**Plot**	**5**	**-506.6**	**0.00**
sTemp	Plot	4	-466.0	40.61
- (Null model)	Plot	3	-468.1	38.43
